# TiO_2_–Carbon Nanotube Hybrid Gas Sensors: From Interfacial Mechanisms and Architectures to Performance Benchmarking and Design Rules

**DOI:** 10.3390/s26196009

**Published:** 2026-09-23

**Authors:** Marciano Sánchez Tizapa, Miriam Tostado Plascencia, Alejandra Carreon-Alvarez, Héctor Huerta Ávila, Rocío Castañeda Valderrama

**Affiliations:** 1 Departamento de Ciencias Naturales y Exactas, Centro Universitario de los Valles, Universidad de Guadalajara, Carretera Guadalajara–Ameca Km 45.5, Ameca C.P. 46600, Jalisco, Mexico; miriam.tostado@academicos.udg.mx (M.T.P.); maria.carreon@academicos.udg.mx (A.C.-A.); rocio.cvalderrama@academicos.udg.mx (R.C.V.); 2 Departamento de Ciencias Computacionales e Ingenierías, Centro Universitario de los Valles, Universidad de Guadalajara, Carretera Guadalajara–Ameca Km 45.5, Ameca C.P. 46600, Jalisco, Mexico; hector.huerta@valles.udg.mx

**Keywords:** TiO_2_, carbon nanotubes, gas sensors, chemiresistive sensors, heterojunctions, interfacial charge transfer, room-temperature sensing, photoactivation, volatile organic compounds, sensor benchmarking

## Abstract

TiO_2_–carbon nanotube (TiO_2_–CNT) hybrids are promising gas-sensor platforms because they combine the chemically active and photoresponsive surface of TiO_2_ with the high conductivity and interfacial sensitivity of CNT networks. Yet cross-study comparison remains difficult because materials, device architectures, humidity, activation modes, and response definitions vary widely. This comprehensive review connects interfacial mechanisms and architectures with performance benchmarking and design rules. Direct gas-sensing evidence is distinguished from supporting interfacial studies and contextual analogues. We assess band alignment, oxygen vacancies, CNT functionalization, shell thickness, percolation, photoactivation, humidity, and analyte-specific behavior across NO_*x*_, NH_3_, H_2_, H_2_S, VOCs, LPG, O_2_, and humidity sensing. The evidence shows that response is governed more strongly by interface continuity, spatial location, and the dominant current pathway than by nominal composition alone. Thin conformal coatings, discrete oxide decoration, and interpenetrating networks can each be advantageous; none is universally superior. Because response definitions and protocols remain inconsistent, we propose a benchmarking framework separating response magnitude from sensitivity, kinetics, selectivity, stability, humidity robustness, and activation requirements. These relationships yield practical design rules for controlling receptor chemistry, interface quality, percolation, activation energy, and validation conditions, providing a route from proof-of-concept demonstrations toward reproducible, low-power, application-relevant TiO_2_–CNT gas sensors.

## 1. Abbreviations and Symbols

The notation used throughout the review is summarized in Table 1.

Throughout the prose, TiO_2_–CNT is used as the standard generic notation for the hybrid material family and its interface. Slash notation (for example, Pt/TiO_2_/CNT or TiO_2_/MWCNT) is retained only when reproducing a study-specific composition, layer sequence, or material designation for which the slash carries descriptive meaning.

## 2. Review Organization

The review is organized as a sequence of interconnected themes. Following the definition of its scope and literature-selection criteria, the physicochemical foundations of TiO_2_–CNT interfaces are examined, including band alignment, surface oxygen chemistry, defect-mediated adsorption, and interfacial charge transport. The principal synthesis and assembly strategies are then compared in terms of their ability to control oxide morphology, CNT functionalization, interface quality, and transport topology. Subsequent sections establish structure–performance relationships, normalize the diverse response metrics used in the literature, and critically assess sensing performance by analyte class. Cross-analyte design principles and thermal, optical, and room-temperature activation strategies are subsequently discussed. The review concludes by identifying methodological limitations, proposing practical design guidelines, and outlining priorities for reproducible and application-oriented TiO_2_–CNT gas sensors.

## 3. Introduction

Gas-sensor development increasingly requires progression from materials discovery toward mechanism-resolved, architecture-controlled, and quantitatively benchmarked devices. This need spans environmental monitoring, industrial safety, indoor air-quality control, process analytics, medical diagnostics, food preservation, and emerging wearable electronics. The demand for low-power, sensitive, selective, and miniaturizable sensors has therefore driven sustained research into semiconducting oxides, carbon nanomaterials, and hybrid nanostructures capable of transducing gas–solid interactions into robust electrical or optical signals [1,2,3,4,5]. Among metal oxides, titanium dioxide (TiO_2_) has attracted long-standing interest due to its chemical stability, low cost, biosafety, rich polymorphism, photoactivity, controllable defect structure, and compatibility with diverse synthetic routes [1,2,4]. Nevertheless, pristine TiO_2_ also presents well-known limitations when used as a gas-sensitive material, including modest electrical conductivity, incomplete recovery under mild conditions, sluggish kinetics at room temperature, and, in many cases, the need for thermal activation to achieve satisfactory response levels [1,3,5].

Carbon nanotubes (CNTs), by contrast, offer high aspect ratio, low percolation threshold, excellent electrical conductivity, mechanical robustness, and strong sensitivity to local charge perturbations, making them attractive components for low-temperature gas-sensing platforms [3,6,7,8]. However, their sensing behavior depends strongly on chirality, defect density, bundle morphology, purification history, surface functionalization, and junction topology, and bare CNT networks often suffer from cross-sensitivity, signal drift, limited selectivity, and strong water interference in complex atmospheres [5,6,7,8]. The combination of TiO_2_ with CNTs has therefore emerged as an appealing strategy to couple the chemically active and defect-mediated surface chemistry of the oxide with the efficient charge-transport characteristics of the carbon scaffold [9,10,11,12,13].

The appeal of TiO_2_–CNT hybrids lies not merely in the coexistence of two functional materials, but in the interfacial phenomena that emerge once they are coupled at the nanoscale [9,10,11,12,13]. Depending on the architecture, TiO_2_ nanoparticles, nanotubes, nanorods, nanowires, thin conformal shells, or hierarchical oxide domains may decorate, infiltrate, wrap, or bridge CNT frameworks to create mixed transport pathways in which adsorption, carrier depletion, carrier extraction, tunneling, and barrier modulation become spatially distributed processes [11,14,15,16]. Under such conditions, the device no longer behaves as a pure oxide chemiresistor or a pure CNT chemiresistor; instead, its signal reflects an interplay between TiO_2_ surface chemistry, CNT network conduction, TiO_2_–CNT junction energetics, CNT–CNT contact barriers, and electrode interfaces [6,7,8,17,18].

Over the past decade, a wide variety of TiO_2_–CNT-based gas-sensing systems has been reported, including TiO_2_-decorated SWCNT and MWCNT films, interlaced CNT/TiO_2_ flexible structures, TiO_2_ nanotube/CNT hybrids, core–shell coatings obtained by hybrid CVD-ALD, hydrothermally assembled porous networks, photoactivated ternary composites, and array-type platforms employing signal processing or machine-learning-assisted gas discrimination [7,8,15,16,19,20]. These systems have been investigated for NO_2_, NO, NH_3_, H_2_, H_2_S, formaldehyde, acetone, toluene, mixed VOCs, humidity, and oxygen-related sensing [6,7,21,22,23,24,25,26]. Nevertheless, the literature remains fragmented. Sensor responses are reported under different humidity conditions, activation modes, operating temperatures, flow protocols, analyte concentrations, and response definitions, making direct comparison difficult. Mechanistic interpretation is often inferred from performance trends alone, with limited operando evidence for the actual role of the interface, oxygen vacancies, CNT functionalization, or photoinduced charge redistribution [3,5].

Several review articles have already covered TiO_2_ nanomaterials for sensing, TiO_2_-based heterostructures, or TiO_2_ combined with noble metals and carbon materials in broader contexts [1,2,3,4,5]. However, a focused comprehensive review centered specifically on TiO_2_–CNT hybrid composites for gas sensing remains timely for several reasons. First, the field has now matured to the point where recurring design patterns are clearly visible, including partial versus conformal oxide coating, the role of CNT pretreatment, the importance of TiO_2_ crystallinity, and the advantages of light-assisted operation. Second, a sufficiently broad body of analyte-specific studies is now available to extract analyte-dependent structure–performance rules rather than merely summarize isolated case studies. Third, recent contributions on flexible devices, visible-light activation, humidity-tolerant sensing, e-nose platforms, and practical application scenarios have started to shift the field from proof-of-concept materials toward functional sensing systems [5,8,24,27].

In this review, we therefore examine TiO_2_–CNT hybrid composites through a unifying interfacial lens. Rather than treating the hybrid as a simple additive combination, we consider the TiO_2_–CNT interface to be the primary functional unit that governs adsorption, charge separation, electronic transduction, signal amplification, and recovery. Accordingly, the central contribution is not only to summarize reported TiO_2_–CNT gas-sensing performance, but to establish a unified framework connecting interfacial architecture, activation conditions, transport mechanisms, analyte chemistry, and experimentally observed sensor response. The review is therefore organized as a progression from interfacial mechanisms and architectures to performance benchmarking and actionable design rules.

### Scope and Literature Selection Strategy

This article is a comprehensive, structured narrative review rather than a formal systematic review or meta-analysis. The literature coverage emphasizes recent work from 2019–2026 while retaining foundational direct gas-sensing studies from 2013 onward, including Pt/TiO_2_/CNT hydrogen sensing, ALD TiO_2_-coated CNT sensing of NO_2_/O_2_, and ALD TiO_2_/MWCNT ammonia sensing [28,29,30]. The coverage is intentionally broader than the direct sensor corpus because mechanistic interpretation of TiO_2_–CNT devices often depends on evidence from interfacial, photocatalytic, photoelectrochemical, and transport studies.

The literature search was conducted using Google Scholar. Boolean queries were organized around the main themes of the review. Representative search strings included TiO_2_ AND “carbon nanotube” AND “gas sensor” for the core topic; (“titanium dioxide” OR TiO_2_) AND (“carbon nanotube” OR CNT) AND (review OR “state of the art”) for general reviews; TiO_2_ AND CNT AND (sol-gel OR hydrothermal OR electrospinning OR ALD OR CVD) for synthesis routes; TiO_2_ AND CNT AND (heterojunction OR “charge transfer” OR interface) for interfacial mechanisms; TiO_2_ AND CNT AND (“room temperature” OR ambient) for low-temperature operation; TiO_2_ AND CNT AND (photoactivated OR UV OR “visible light”) for photoactivation; and TiO_2_ AND CNT AND (stability OR humidity OR reproducibility) for reliability-related studies. Analyte-specific searches were also performed, including TiO_2_ AND CNT AND NO_2_ and TiO_2_ AND CNT AND NH_3_, with broader synonym-based combinations used when needed to capture alternative terminology for TiO_2_, CNTs, ammonia, and chemiresistive gas sensing.

The resulting literature corpus was organized in a comparative screening matrix containing 154 articles. Studies were classified according to their relevance to the central scope of the review into 30 core studies, 63 supporting studies, and 61 peripheral studies. Core studies comprised direct TiO_2_–CNT gas-sensing investigations and closely related methodological reviews, whereas supporting studies were retained for synthesis, interfacial-mechanism, materials comparison, or contextual discussion. Peripheral studies, mainly focused on photocatalysis, energy, remediation, aqueous systems, solar cells, or non-gas sensing, were not used as primary evidence for the central analysis. A second screening step prioritized 25 articles for detailed discussion and 21 additional studies for introduction, context, and mechanistic support.

Search results were screened for relevance to TiO_2_–CNT gas sensing, interfacial mechanisms, device architecture, operating conditions, and performance evaluation. Rather than reporting a PRISMA-style record count that cannot be reconstructed reliably from the present bibliographic archive, the review applies an explicit evidential hierarchy. Core evidence consists of TiO_2_–CNT or TiO_2_–CNT-containing gas sensors with an identifiable analyte, operating condition, and transduction signal. Supporting evidence is used when a TiO_2_–CNT study clarifies oxide nucleation, shell continuity, CNT functionalization, charge extraction, defect chemistry, or loading effects. Contextual evidence from other TiO_2_–carbon or metal-oxide/carbon systems is retained only when it tests a transferable mechanism or defines a useful boundary condition.

The distinction is important because TiO_2_–CNT gas-sensing papers frequently borrow mechanistic language from photocatalysis and photoelectrochemistry. Charge separation demonstrated in a photocatalyst, for example, does not by itself establish that the same pathway controls the resistance of a chemiresistor. Throughout this review, mechanistic statements are therefore weighted according to the type of experiment that supports them, and quantitative performance comparisons are restricted to studies with sufficiently explicit response definitions and operating conditions.

Generative AI-assisted tools were used during literature screening and manuscript preparation to support language refinement, structural organization, literature triage, and drafting of conceptual synthesis graphics. They were not used as a source of scientific evidence or to generate experimental data. All bibliographic entries, numerical values, mechanistic claims, inclusion decisions, interpretations, and visual content were checked and edited by the authors against the cited literature.

Table 2 summarizes the evidential hierarchy used to avoid assigning the same weight to direct sensor reports and contextual analogies. The remainder of the review follows this logic by connecting evidence selection, interfacial interpretation, analyte-specific benchmarking, and falsifiable design rules.

To make this hierarchy visible throughout the review, the strength of the wording is matched to the strength of the evidence. Direct TiO_2_–CNT gas-sensor experiments are described as demonstrating or showing a sensing relationship; TiO_2_–CNT studies from adjacent interfacial contexts are described as supporting or being consistent with a proposed mechanism; and contextual analogues are used only to suggest a testable hypothesis or boundary condition. In particular, an analogue is not used by itself to establish response polarity, sensitivity, selectivity, detection limit, or recovery kinetics for a TiO_2_–CNT gas sensor.

## 4. TiO_2_–CNT Interfaces and Gas-Sensing Transduction

### 4.1. Why the TiO_2_–CNT Interface Is the Real Sensing Unit

The central scientific value of TiO_2_–CNT hybrids for gas sensing lies in the fact that the two components are not merely juxtaposed, but electronically and chemically coupled in a way that redistributes both adsorption and transport processes. TiO_2_ contributes chemically active adsorption sites, oxygen-vacancy-mediated surface reactivity, analyte-sensitive modulation of electron density, and, under irradiation, photoinduced carrier generation. CNTs contribute long-range conductive pathways, low percolation thresholds, structural anisotropy, local electric-field concentration at junctions, and strong sensitivity to charge transfer from neighboring phases [9,10,11,12]. Once these phases are integrated, the resulting sensing layer no longer behaves like either pristine TiO_2_ or pristine CNTs, but as a distributed interfacial network in which oxide grains, oxide surface states, CNT sidewalls, CNT defects, CNT–CNT junctions, TiO_2_–CNT contacts, and metal electrodes all participate in the measured response.

This perspective is essential because nominal composition alone is often a poor predictor of sensor performance. Two TiO_2_–CNT composites with similar oxide loading may exhibit sharply different response magnitude, polarity, kinetics, and humidity tolerance if they differ in CNT pretreatment, oxide shell continuity, nanoparticle spacing, crystallinity, or interfacial bonding. For a comprehensive review, therefore, the TiO_2_–CNT interface must be treated as the real sensing unit, whereas composition becomes only one of several structural descriptors needed to rationalize performance.

### 4.2. Electronic Structure and Complementary Roles of TiO_2_ and CNTs

TiO_2_ is a wide-band-gap semiconductor whose gas-sensing behavior is generally linked to surface adsorption of oxygen, band bending near the surface, depletion-layer formation, and analyte-dependent modulation of carrier concentration [1,2,5]. Its response depends on polymorph, crystallinity, exposed facets, defect density, particle size, and hydroxylation state. Anatase is frequently preferred in sensing-related studies because it combines relatively high surface area, rich defect chemistry, and compatibility with low-temperature processing, whereas rutile can contribute different transport properties and different adsorption energetics. Mixed-phase systems may introduce additional interfaces that modify local carrier dynamics and sometimes enhance response through internal heterojunction effects [1,4].

CNTs, whether single-walled or multi-walled, behave as quasi-one-dimensional conductive nanostructures whose practical transport properties in sensing layers are governed less by the ideal electronic character of isolated nanotubes than by collective conduction through a disordered ensemble of nanotube segments, bundle contacts, and intertube junctions [3,6,7,8]. Depending on chirality, CNTs may be metallic or semiconducting, but in most experimentally relevant films the measurable current is controlled by percolation through multiple contact barriers and defect-mediated pathways rather than by the intrinsic conductance of a single nanotube [6,8,9,12]. In TiO_2_–CNT hybrids, CNTs therefore play a dual role: they lower the baseline resistance of the sensing layer and also provide efficient pathways for the redistribution or extraction of charges generated at TiO_2_ surfaces during adsorption or photoactivation [9,10,11,13].

Functionally, TiO_2_ tends to dominate the receptor role by providing analyte-sensitive surface chemistry, whereas CNTs tend to dominate the transducer role through network-level resistance modulation. However, this distinction should not be overstated. Functionalized CNTs may also directly bind polar analytes and water, while thick or continuous oxide coatings may force the device into an oxide-dominated transport regime. Thus, the best-performing hybrids are not those in which one component dominates absolutely, but those in which receptor and transducer functions overlap constructively.

### 4.3. Band Alignment, Contact Formation, and Interfacial Charge Transfer

When TiO_2_ is coupled with CNTs, electronic equilibration occurs across the oxide–carbon interface, giving rise to charge redistribution, band bending in the semiconducting phase, and modified interfacial barrier heights [9,10,11,12,13]. In a simplified description, TiO_2_ behaves as an n-type semiconductor, whereas CNT networks act as conductive or semiconductive carbon phases whose effective work function depends on chirality, defect density, purification history, and surface functionalization [9,13,31]. The resulting interfacial energetics govern how adsorption-induced perturbations at TiO_2_ surfaces are transmitted to the conductive scaffold and ultimately converted into measurable changes in device resistance [9,10,11,31]. In many TiO_2_–CNT systems, CNTs are therefore interpreted as charge-accepting or charge-extracting pathways, especially under photoactivated conditions, although the actual direction and efficiency of transfer depend strongly on local bonding, defect chemistry, and structural heterogeneity at the interface [9,10,13,31].

Before contact, TiO_2_ and CNTs possess distinct Fermi levels. After contact, electrons may be redistributed until equilibrium is reached, and the resulting local electronic structure determines how adsorption-induced charge perturbations are transmitted through the device. In many discussions, CNTs are treated as electron acceptors or extraction pathways, especially in photoactivated systems, where they are assumed to reduce recombination and facilitate current transport [10,11,13]. This interpretation is qualitatively reasonable, but the real situation is usually more complex because CNTs may carry oxygen-containing surface groups, residual catalyst particles, structural defects, and variable contact barriers that render the junction population highly heterogeneous.

A particularly useful atomistic reference is the combined experimental–DFT study of Borbón-Núñez et al., who showed that oxygen-containing carbon groups can strongly affect TiO_2_ nucleation and interfacial stability during ALD growth [32]. Although the study was not a gas-sensor report, its value here is specific: carboxylated carbon surfaces promoted stronger TiO_2_ incorporation and more stable oxide–carbon interaction than hydroxylated or aldehyde-functionalized surfaces. This supports a direct design rule for TiO_2_–CNT sensors: CNT functionalization should be optimized as an interfacial nucleation and electronic-coupling variable, not treated merely as a dispersion step.

The sensing consequence of this band alignment is profound. Gas adsorption on TiO_2_ is no longer translated only through oxide grain-boundary resistance. Instead, it may alter the interfacial potential at TiO_2_–CNT contacts, modify hopping and tunneling across CNT-rich pathways, and shift the relative contribution of oxide-rich and carbon-rich regions to the total device resistance. This helps explain why superficially similar TiO_2_–CNT systems can exhibit very different response amplitudes, recovery rates, and analyte selectivities.

This interfacial equilibration is incorporated into the multiscale transduction framework in Figure 1. Luo et al. provide a concise supporting example on CNT scaffold engineering before TiO_2_ deposition [33]. Their TiO_2_/sheared-CNT architecture shows that partial nanotube unzipping can create anchoring sites for small anatase domains while preserving conductive CNT backbones. For gas sensing, the transferable design rule is clear: defect engineering should create sufficient nucleation and adsorption sites without destroying percolative electronic transport.

### 4.4. Surface Chemistry, Oxygen Vacancies, and Ionosorbed Oxygen

The surface chemistry of TiO_2_–CNT hybrids is governed by several classes of active sites, including oxygen vacancies on TiO_2_, hydroxyl groups, coordinatively unsaturated titanium centers, residual precursor-derived defects, CNT sidewall defects, oxygen-containing functional groups on CNTs, and mixed TiO_2_–CNT interfacial regions. Among these, oxygen vacancies are especially important because they influence both adsorption energetics and the local electronic structure [9,34,35]. Vacancy-rich TiO_2_ surfaces can alter oxygen adsorption, change trapped-carrier populations, and promote analyte-specific interaction pathways. CNT functionalization, in turn, determines how effectively oxide nuclei anchor to the nanotube surface and how much direct analyte interaction occurs on the carbon component.

In air, oxygen adsorption on TiO_2_ generally withdraws electrons from the oxide and forms ionosorbed oxygen species, producing an electron-depleted near-surface region that governs the baseline resistance of n-type oxide sensors [1,2,3,5]. At relatively low temperature, $O_{2}^{-}$ is commonly regarded as the dominant adsorbate, whereas $O^{-}$ becomes increasingly relevant at higher temperatures [1,5]. The simplified adsorption sequence can be written as

(1) $$\begin{array}{cc} O_{2}\left(g\right) & ⇌O_{2}\left(\mathrm{ads}\right) \end{array}$$

(2) $$\begin{array}{cc} O_{2}\left(\mathrm{ads}\right)+e^{-} & ⇌O_{2}^{-}\left(\mathrm{ads}\right) \end{array}$$

(3) $$\begin{array}{cc} O_{2}^{-}\left(\mathrm{ads}\right)+e^{-} & ⇌2O^{-}\left(\mathrm{ads}\right) \end{array}$$

These reactions generate an electron-depleted near-surface region in TiO_2_. When the oxide is nanostructured, the depletion region may affect a substantial fraction of the particle volume [1,5]. In TiO_2_–CNT hybrids, however, this process does not merely increase oxide resistance. The altered surface potential may also modulate the junction potential at TiO_2_–CNT contacts and redistribute current through the conductive carbon scaffold [9,10,11,12].

For reducing gases, reaction with ionosorbed oxygen can release electrons back into the sensing layer, decreasing resistance in oxide-dominated conduction paths [1,2,3,5]:

(4) $$\mathrm{Reducing}\;\mathrm{gas}+O^{-}\left(\mathrm{ads}\right)\to \mathrm{Oxidized}\;\mathrm{products}+e^{-}.$$

For oxidizing gases such as NO_2_, direct electron withdrawal may intensify depletion by extracting electrons from the sensing layer or by forming adsorbed ionic species [1,2,5]:

(5) $${\mathrm{NO}}_{2}\left(g\right)+e^{-}\to {\mathrm{NO}}_{2}^{-}\left(\mathrm{ads}\right).$$

In TiO_2_–CNT composites, these oxide-centered expressions remain useful as a first approximation, but the actual transduction pathway depends on where the dominant resistance resides, namely oxide grains, oxide grain boundaries, TiO_2_–CNT contacts, CNT–CNT junctions, or electrode interfaces [8,9,11,12,17].

### 4.5. Role of CNT Functionalization and Defect Engineering

CNT functionalization is often introduced as a pretreatment step to improve oxide anchoring or dispersion, but it has much deeper mechanistic consequences. Oxidative treatments create carboxyl, hydroxyl, carbonyl, and epoxide groups that improve wettability and facilitate TiO_2_ nucleation. At the same time, they create defects that alter intrinsic CNT transport and introduce additional adsorption sites for polar molecules and water. In sensing terms, functionalization influences at least four variables simultaneously: oxide coverage uniformity, CNT baseline resistance, direct analyte affinity at the carbon surface, and the effective work function of the CNT network [7,13,31].

A moderately functionalized CNT scaffold may provide a useful compromise between oxide anchoring and conductive continuity [7,13,32]. Excessive oxidation, however, can fragment the conductive network, increase electrical resistance, and enhance water adsorption, potentially making the device more sensitive to humidity than to the target gas [7,13,36]. This is particularly relevant when comparing acid-treated CNTs, plasma-treated CNTs, shortened CNTs, and commercially pre-functionalized nanotubes. Even when the nominal TiO_2_–CNT ratio is similar, differences in CNT pretreatment can produce markedly different sensing behavior [7,32,36].

### 4.6. Depletion Layers, Percolation, and Network-Level Transduction

A rigorous treatment of TiO_2_–CNT gas sensing must go beyond the classical single-grain oxide model and incorporate percolation concepts. In practical devices, current does not flow through a perfectly uniform film but through a disordered network composed of oxide grains, oxide grain boundaries, CNT segments, CNT–CNT junctions, TiO_2_–CNT contacts, and metal-film interfaces. The total device resistance is therefore an emergent property of network topology rather than a simple sum of component resistances.

This has two major consequences. First, the same chemical event may produce different electrical signatures depending on where the current is concentrated. If current flows mainly through CNT-rich pathways, adsorption at a relatively small number of strategically located TiO_2_ junctions may be sufficient to produce a strong signal. If current is forced through thick oxide-rich domains, the response may become larger but slower and noisier. Second, there is usually an architectural optimum rather than a monotonic dependence on oxide content. Too little oxide yields a CNT-dominated device with limited selectivity. Too much oxide suppresses percolation and compromises transport. The best systems therefore sit near a balance point where conductive continuity and active surface density are preserved simultaneously [14,16,17,18].

### 4.7. Humidity as a First-Order Interfacial Variable

Humidity is one of the most underestimated yet decisive variables in TiO_2_–CNT gas sensing. Water competes for TiO_2_ adsorption sites, alters the population of ionosorbed oxygen species, changes proton availability, and modifies interfacial dipoles. On CNTs, particularly oxidized CNTs, water affects direct adsorption and tunneling at junctions. Consequently, humidity may alter baseline resistance, response amplitude, recovery kinetics, and in some cases even the apparent sign of the response [7,26,37].

The importance of humidity is amplified in TiO_2_–CNT hybrids because both components are water-sensitive. TiO_2_ favors hydroxylation and proton-coupled surface chemistry, whereas functionalized CNTs strongly interact with water through oxygen-containing groups. Humidity should therefore not be treated as secondary metadata, but as an interfacial design variable that can destabilize the sensor or, in some cases, be deliberately exploited for humidity sensing.

Four humidity-induced distortion pathways are especially important to distinguish: competitive occupation of TiO_2_ adsorption sites, humidity-dependent hydroxylation and surface oxygen chemistry, protonic conduction through adsorbed water layers, and modulation of CNT junctions or work function by water adsorption. These pathways can change baseline resistance, analyte response, and apparent selectivity without any change in the intrinsic affinity of the target gas. Among the direct TiO_2_–CNT evidence, Hwang et al. addressed severe water interference in breath-oriented acetone sensing by adding upstream dehumidification [7], whereas the humidity-sensing studies of Kumar et al. and Estefani et al. show why water itself can become a dominant transduction variable [26,37]. Beyond dehumidification, the practical mitigation strategies proposed here include humidity-conditioned calibration, a colocated RH reference channel, selective or hydrophobic inlet layers, and temperature- or light-modulated measurements when compatible with the application. These latter strategies should be validated experimentally for each TiO_2_–CNT architecture rather than assumed to be universally effective.

### 4.8. Photoactivation and Interfacial Carrier Dynamics

Photoactivation adds another layer of mechanistic richness to TiO_2_–CNT hybrids. Upon irradiation with photons of sufficient energy, TiO_2_ generates electron–hole pairs according to

(6) $${\mathrm{TiO}}_{2}+h\nu \to e^{-}+h^{+}.$$

These carriers may participate in surface redox chemistry, modify the population of ionosorbed oxygen species, and improve desorption and recovery. When CNTs are coupled to TiO_2_, they are often invoked as rapid electron-transport channels that reduce recombination and preserve useful charge separation [13,19,24,25,38]. Whether this interpretation is quantitatively valid in every architecture remains open, but it captures a major reason why light-assisted TiO_2_–CNT sensing is attractive: it enables low-temperature operation without discarding oxide-based receptor chemistry.

The mechanistic caution is reinforced by the intensity-modulated photocurrent spectroscopy study of Peón-Díaz et al., who systematically varied the CNT content in CNTs/TiO_2_ photoelectrodes and showed that performance is not governed by a simple “more CNTs equals faster electron extraction” rule [39]. Their results indicate that the optimum response depends on a balance among CNT loading, TiO_2_ overlayer thickness, CNT chemical capacitance, hole-transfer efficiency, and interfacial recombination. In particular, the best CNTs/TiO_2_ composites changed the collection regime from trap-limited diffusion in TiO_2_ to more direct charge extraction, whereas excessive CNT content and too thin a TiO_2_ layer increased the probability of hole trapping and recombination at the CNTs/TiO_2_ interface [39]. For the present review, this study is classified as supporting interfacial evidence rather than direct gas-sensor evidence. It demonstrates how CNT loading, TiO_2_ overlayer thickness, charge extraction, and recombination can interact in a photoelectrochemical TiO_2_–CNT architecture, but it does not establish the response sign, sensitivity, selectivity, or kinetics of a gas sensor. The result is therefore used only to motivate testable hypotheses about light-assisted charge collection and recombination in TiO_2_–CNT sensing.

Photoactivated sensing becomes especially important for weakly adsorbing VOCs, dye-sensitized platforms, visible-light-assisted systems, and devices whose recovery would otherwise remain poor at room temperature. In such cases, the TiO_2_–CNT interface is not merely a passive contact but a dynamic photochemical interface whose carrier lifetime and extraction pathways directly influence sensing performance.

### 4.9. Working Hypotheses for Structure–Performance Interpretation

From the discussion above, several working hypotheses emerge and will guide the remainder of this review. Figure 1 integrates the gas-phase, surface, interfacial, network, and recovery processes that determine the measured signal, whereas Table 3 condenses the principal physicochemical variables that control these regimes. First, high sensing performance is more likely when TiO_2_ is spatially distributed so that active adsorption sites are abundant but CNT percolation remains intact. Second, CNT functionalization improves oxide integration but may intensify humidity sensitivity and transport disorder if excessive. Third, the dominant resistance-modulation pathway is architecture-dependent and cannot be inferred from oxide polarity alone. Fourth, light activation can fundamentally alter the transduction mechanism rather than simply accelerate recovery. Fifth, reproducible sensing requires not only compositional control but also control of interface continuity, shell thickness, CNT pretreatment, and environmental water content.

These hypotheses can be condensed into three limiting transduction regimes. In an oxide-dominated regime, gas adsorption primarily modulates TiO_2_ depletion layers and grain boundaries. In a CNT-dominated regime, the signal is governed mainly by CNT–CNT junctions and direct adsorption on functionalized carbon. In the most useful hybrid regime, adsorption at TiO_2_ or mixed TiO_2_–CNT sites is amplified by a still-percolated CNT network. A major objective of rational design is therefore not to maximize one phase, but to place the device inside this interfacial amplification window.

## 5. Synthesis, Assembly, and Sensor Architecture

The performance of TiO_2_–CNT gas sensors is inseparable from the route by which the hybrid layer is created. The synthesis route controls not only morphology and crystallinity, but also shell continuity, interfacial bonding, active surface accessibility, porosity, percolation topology, and the degree of damage or preservation of the CNT scaffold. For this reason, synthesis must not be viewed as a routine preparative detail. In TiO_2_–CNT systems, synthesis is itself a design parameter that frequently determines whether the resulting hybrid behaves as a true heterointerface-based sensing material or as a poorly integrated physical mixture [10,11,12,15,40].

### 5.1. Sol–Gel and Related Wet-Chemical Routes

Sol–gel processing remains one of the most common approaches for fabricating TiO_2_–CNT composites because it offers compositional flexibility, relatively low equipment cost, and compatibility with coatings, powders, and thin films. In typical sol–gel routes, titanium alkoxide precursors undergo hydrolysis and condensation in the presence of CNTs, which may be pristine, oxidized, shortened, or otherwise functionalized. Depending on precursor chemistry, CNT pretreatment, and processing conditions, the resulting materials may consist of TiO_2_ nanoparticles decorating CNT bundles, disordered oxide coatings on CNT walls, or mixed agglomerates in which the oxide and carbon phases are intimately but not necessarily uniformly distributed [31,41]. The principal advantages of sol–gel processing are its simplicity, versatility, and potential scalability, while its applicability extends beyond conventional sensing layers to the formation of porous oxide coatings on CNT-supported architectures.

A foundational sensor study by Santangelo et al. demonstrates why sol–gel processing is especially informative when composition is varied systematically [28]. Pt/TiO_2_/CNT nanohybrids containing 2 wt% Pt were prepared from sol–gel TiO_2_/CNT composites and used as resistive H_2_ sensors near room temperature in an inert carrier. By varying the CNT fraction over a broad range, the authors showed that responsiveness was not monotonic with carbon loading and identified an optimum near 34 wt% CNT. Their material characterization linked the response to TiO_2_ stoichiometry, oxide agglomeration and the extent of TiO_2_–CNT contact, supporting an oxide-mediated electron-transfer mechanism in which the CNT network acts as the electrical transducer [28]. This work is important for the present review because it provides direct experimental evidence that sol–gel TiO_2_–CNT sensing is governed by a coupled composition–morphology–interface optimum rather than by maximum CNT content.

A structurally focused sol–gel contribution by Grozdanov and co-workers is especially useful for separating nominal composition from true interfacial modification [42]. In that study, TiO_2_/CNT nanocomposites were prepared from titanium tetraisopropoxide in ethanol with acid hydrolysis, followed by calcination at 400 °C; the authors compared activated MWCNTs-COOH, SWCNTs, different MWCNT contents, and Pt- or Co-containing ternary compositions [42]. The main value of the work for gas-sensor design is mechanistic rather than performance-based: XRD showed anatase formation with peak broadening and crystallite-size reduction after CNT incorporation, Raman spectra showed red shifts of TiO_2_ modes together with shifts of CNT D/G bands, and SEM indicated that SWCNTs or higher MWCNT loading improved the physical interlocking of the carbon scaffold within the TiO_2_ matrix [42]. These observations support the view that wet-chemical TiO_2_–CNT synthesis can generate lattice expansion, Ti–O–C-related coupling, smaller oxide domains, and a more disordered or non-stoichiometric anatase phase, all of which are relevant to sensor behavior because they modify oxygen-vacancy density, adsorption-site distribution, and interfacial charge-transfer pathways. At the same time, the study should be interpreted as a structural benchmark rather than a complete sensing benchmark, since it does not provide systematic chemiresistive response metrics; its importance lies in showing how CNT type, CNT loading, and metallic additives can reshape the oxide phase before the material is incorporated into a device [42].

### 5.2. Hydrothermal and Solvothermal Strategies

Hydrothermal and solvothermal methods are especially useful when the target architecture involves TiO_2_ nanotubes, nanorods, nanosheets, hierarchical oxide morphologies, or strongly anchored oxide domains on carbon scaffolds. These methods enable good morphological control through temperature, pH, mineralizers, precursor concentration, and reaction time. They also frequently yield improved crystallinity and stronger oxide-carbon integration compared with purely ambient-temperature wet chemistry [4,40].

For gas sensing, hydrothermal routes are attractive because they can produce porous, open, and high-surface-area TiO_2_ architectures that favor gas diffusion and increase the accessible receptor area [4,40]. However, these advantages depend strongly on the dispersion and relative loading of the carbon phase. In hydrothermally prepared TiO_2_/MWCNT composites, excessive CNT loading can promote clustering and reduce accessible surface area, pore volume, and TiO_2_ exposure [43]. CNT pretreatment and shortening can also modify TiO_2_–CNT interfacial contact and charge-transfer behavior [13]. Consequently, materials prepared under nominally similar hydrothermal conditions may exhibit substantially different morphologies and interfacial properties when CNT pretreatment, dispersion, or loading differs.

Everhart et al. provide a concise warning about CNT loading in hydrothermally prepared TiO_2_/MWCNT photocatalysts [43]. In gas-phase acetaldehyde degradation, 1 wt% MWCNTs nearly doubled the apparent rate constant relative to TiO_2_, whereas 5 wt% MWCNTs performed worse than the oxide alone because CNT clustering reduced surface area, pore volume, and effective TiO_2_ exposure. Because this is a photocatalytic rather than a gas-sensor experiment, it is used here only as supporting evidence for a non-monotonic loading effect. It establishes that carbon excess can reduce accessible TiO_2_ area through clustering, light screening, and morphological changes, but it does not by itself establish how CNT loading changes chemiresistive response [43].

The packed-bed microfluidic TiO_2_/CNT and TiO_2_/AuCNT systems reported by Farah et al. add an architectural lesson that complements powder and film syntheses [44]. In their work, the entangled CNT network was not merely a conductive additive; it enabled physical retention of the TiO_2_-based hybrid as a porous catalytic bed in a microfluidic photoreactor, allowing continuous flow operation without observable TiO_2_ or Au leaching. This is relevant to sensor fabrication because it shows that CNT topology can serve simultaneously as an electronic pathway, an interfacial charge-separation scaffold, and a mechanically useful support for structuring photoactive TiO_2_ domains in confined geometries [44].

### 5.3. Atomic Layer Deposition and Other Interface-Controlled Approaches

Atomic layer deposition (ALD) is among the most powerful tools for constructing TiO_2_–CNT heterostructures with high interfacial precision. Because ALD relies on self-limiting surface reactions, it enables near-conformal deposition of TiO_2_ with nanometer-scale thickness control, even on high-aspect-ratio carbon scaffolds. This makes it ideal for systematic studies of shell continuity, thickness, crystallization, and interfacial bonding [10,12,15,32]. In the context of gas sensing, ALD is especially valuable because it allows systematic examination of how much oxide is needed to provide active adsorption without suppressing CNT percolation.

The work of Borbón-Núñez et al. is especially informative for understanding the first ALD cycles on carbon-rich substrates [32]. The authors functionalized nanoporous carbon with ϕ-OH, ϕ-CHO, and ϕ-COOH groups and then exposed the materials to 20 TDMAT/H_2_O ALD cycles. FTIR, Raman, XPS, and TEM confirmed that TiO_2_ nucleation was strongly dependent on the oxygenated surface group: TiO_2_/CM-ϕ-COOH showed the largest titanium incorporation and the most evident surface coverage, whereas hydroxylated and aldehyde-functionalized carbons showed more limited, isolated nucleation. DFT calculations reproduced this trend mechanistically, with adsorption energies following TiO_2_/CM-ϕ-COOH > TiO_2_/CM-ϕ-CHO > TiO_2_/CM-ϕ-OH, and the carboxylated interface showing the most effective charge screening and bonding. For gas-sensing TiO_2_–CNT hybrids, this study provides a useful design rule: controlled oxidation or carboxylation of CNTs before ALD may improve oxide nucleation and interfacial reproducibility, but excessive functionalization must still be balanced against possible degradation of CNT conductivity.

Two earlier direct gas-sensing studies make the thickness issue particularly clear. Marichy et al. used ALD to prepare amorphous TiO_2_ coatings on CNTs for resistive NO_2_ and O_2_ sensing and found that a homogeneous, continuous coating approximately 5.5 nm thick produced a higher response at 150 °C than discontinuous coatings or TiO_2_ nanoparticle decoration [29]. The devices also exhibited p-type response behavior, illustrating that the sign of the sensor signal cannot be inferred from the nominal n-type character of TiO_2_ alone. Kaushik et al. subsequently coated MWCNTs with nominal TiO_2_ thicknesses of 5, 10 and 20 nm for room-temperature NH_3_ sensing and showed the opposite side of the same design problem: the thinnest coatings gave the strongest NH_3_ response, while a carboxyl plasma-polymer interlayer improved TiO_2_ nucleation and increased response relative to untreated CNTs [30]. Taken together, these studies show that “continuous” and “discrete” should not be treated as intrinsically good or bad architectures. What matters is whether the oxide morphology creates sufficient receptor area and barrier modulation while preserving an electrically sensitive CNT network.

A particularly instructive example is the work of Bakos et al., who used ALD to coat hydroxyl-functionalized MWCNTs with single and double oxide layers, generating CNT–TiO_2_, CNT–ZnO, CNT–TiO_2_–ZnO, and CNT–ZnO–TiO_2_ heterostructures with uniform anatase and wurtzite shells [15]. Although the paper spans both photocatalysis and gas sensing, its real value for this review lies in the unusually clean demonstration that shell sequence matters. In their NH_3_ measurements, multilayer structures exhibited stronger responses than single-layer analogues because the thicker oxide shells increased resistivity and amplified resistance modulation, but the response depended decisively on which oxide formed the outer surface. When TiO_2_ was the outer layer, the NH_3_ signal was markedly higher than when ZnO terminated the heterostructure, which the authors attributed to the lower conductivity of TiO_2_ and the correspondingly larger relative resistance change upon gas interaction [15]. This is an important mechanistic lesson for TiO_2_–CNT sensor design: the outermost oxide should not be treated as a passive coating detail, because it determines the chemistry of first contact with the analyte and sets the electrical leverage by which interfacial adsorption is converted into a measurable signal.

The same study also provides a useful caution against overgeneralized architecture claims. Bakos et al. found that the multilayer samples were not universally superior: for photocatalysis, the best behavior was obtained when ZnO was the outer layer, whereas for NH_3_ sensing TiO_2_ termination was advantageous [15]. In other words, the optimal stacking sequence is application-dependent because photocatalysis privileges the surface generation and utilization of reactive charge carriers, while chemiresistive gas sensing privileges analyte-specific adsorption and resistance modulation at the outer shell. For a comprehensive review focused on gas sensors, this distinction is highly valuable because it shows that heterostructure design cannot be transferred uncritically from photocatalysis to sensing, even when the same TiO_2_–CNT platform is used.

Hybrid CVD-ALD approaches have also been used to create porous CNT-TiO_2_ core–shell structures with highly uniform anatase shells. Such architectures are particularly appealing because they maximize interfacial area while preserving a mechanically coherent and electrically connected CNT backbone [11]. Their drawbacks are mainly complexity, cost, and the need for stringent process control, but for high-level mechanistic studies and advanced devices, ALD-based architectures provide some of the cleanest model systems available in the field.

### 5.4. Dielectrophoretic Assembly and Spatially Selective Deposition

Dielectrophoretic assembly offers a distinct advantage in that it allows the CNT network to be positioned first and the oxide phase to be introduced later in a spatially controlled manner. This idea is exemplified by multistep dielectrophoretic routes in which aligned CNTs are first assembled between electrodes and TiO_2_ nanoparticles are subsequently deposited under different electrical conditions [14]. Such strategies are especially informative because they decouple network formation from oxide functionalization. As a result, the conductive scaffold can be preserved while TiO_2_ is placed where it is most beneficial for adsorption and interfacial modulation.

This approach directly addresses one of the recurring problems in TiO_2_–CNT sensing, namely that simultaneous mixing of CNTs and oxide particles often produces uncontrolled aggregate structures. Spatially selective routes, by contrast, provide a clearer relationship between architecture and function and therefore carry special value for both mechanistic studies and room-temperature sensor design.

### 5.5. In Situ Growth, Interlaced Films, and Free-Standing Architectures

Beyond nanoparticle decoration, a growing fraction of the literature has moved toward deliberately engineered architectures such as interlaced films, free-standing membranes, CNT-supported oxide networks, and partial core–shell structures. These designs aim to optimize the competing requirements of conductivity, gas accessibility, surface reactivity, and mechanical stability [16,45,46]. Partial oxide coverage often preserves CNT transport while still providing abundant oxide-active sites, whereas interlaced architectures may physically separate transport-rich and adsorption-rich domains in a mechanically robust film.

The CVD study of Campos Guaglianoni et al. is retained only as a processing caution: CNT growth conditions can improve carbon connectivity while simultaneously altering oxide crystallinity, defect density, nanotube integrity, and interfacial charge extraction [46]. For TiO_2_–CNT gas sensors, this means that CVD-derived architectures require careful separation of morphology, phase composition, and transport effects.

Such architectures are especially important for flexible devices and low-power operation, because they reduce the likelihood that the sensor becomes transport-limited by a thick oxide layer. They also support the broader conclusion that architecture often matters more than nominal composition. A well-organized TiO_2_–CNT network with moderate oxide loading may outperform a more heavily loaded but poorly connected composite.

The synthesis route does not merely determine the presence of TiO_2_ and CNTs, but defines their spatial arrangement, interfacial contact, accessible surface area, and dominant charge-transport pathways. Consequently, apparently similar compositions may exhibit markedly different sensing behavior when assembled as decorated nanotubes, conformal core–shell structures, interpenetrating networks, or hierarchical free-standing films. These principal morphological configurations are compared in Figure 2.

### 5.6. Synthetic Control Versus Sensing Function

A central conclusion emerging from the available synthesis literature is that synthetic precision is most valuable when it directly improves one or more of the following: interfacial continuity, active-site accessibility, gas diffusion pathways, conductive continuity, or environmental robustness. High crystallinity alone does not guarantee better sensing. Nor does greater oxide loading automatically improve response. The challenge is to tune multiple structural variables simultaneously so that adsorption, interfacial charge transfer, and current transport all remain favorable under the intended operating conditions.

## 6. Structure–Performance Relationships in Sensor Design

The TiO_2_–CNT literature indicates that gas-sensing performance is controlled less by the nominal presence of TiO_2_ and CNTs than by how the two phases are spatially, electronically, and chemically integrated. The most relevant structural variables are TiO_2_ phase and crystallinity, oxide loading, particle or shell thickness, CNT type, defect density, functionalization state, network density, porosity, and electrode contact geometry [6,7,14,18,26]. These variables do not act independently. A change that improves one function, such as increasing oxide surface area, may simultaneously damage another function, such as CNT percolation or gas diffusion.

A useful way to interpret TiO_2_–CNT sensors is therefore to separate receptor, transducer, and transport roles. TiO_2_ usually acts as the main receptor phase because it supplies oxygen vacancies, chemisorbed oxygen, hydroxyl groups, and analyte-sensitive surface states. CNTs usually act as the main transducer phase because they provide conductive pathways and amplify local charge perturbations. The interface couples both functions by defining charge redistribution, junction barriers, local depletion regions, photocarrier extraction, and recovery kinetics. Strong performance is achieved only when these three levels remain mutually compatible.

The most recurrent structure–performance rule is non-monotonicity, as summarized in Table 4. Increasing TiO_2_ loading can improve adsorption and enlarge depletion-layer modulation, but excessive oxide coverage raises baseline resistance, blocks CNT junctions, slows recovery, and reduces the electrical leverage of the nanotube scaffold. Similarly, CNT functionalization improves TiO_2_ anchoring and dispersion, but excessive oxidation may introduce trap states, disrupt percolation, or increase humidity sensitivity. Light activation, noble-metal sensitization, and ternary oxides can also be beneficial, but only when they address a specific kinetic or selectivity limitation rather than simply increasing compositional complexity.

In the study-comparison tables below, test temperature, analyte concentration, relative humidity (RH), carrier-gas conditions, and optical activation are reported from the original study wherever available. “NR” is retained only when the corresponding condition is not stated in the source after direct verification; single-concentration studies are identified explicitly as such rather than being represented as concentration ranges. Detection limits are reported separately and are not used as substitutes for the tested concentration range.

### Reconciling Apparently Contradictory Architecture Trends

Direct sensing studies show why architecture cannot be reduced to a universal rule such as “partial decoration is always better” or “more TiO_2_ gives higher response.” Marichy et al. found that an approximately 5.5 nm continuous ALD TiO_2_ coating on CNTs outperformed discontinuous films or nanoparticle decoration for NO_2_/O_2_ sensing at 150 °C [29]. By contrast, Kaushik et al. found an optimum near a 5 nm ALD TiO_2_ coating for room-temperature NH_23_ sensing and showed that both shell thickness and CNT functionalization altered the response [30]. Santangelo et al. observed a non-monotonic H_2_ response with an optimum near 34 wt% CNT in Pt/TiO_2_/CNT hybrids [28], while Ito et al. showed that TiO_2_ nanoparticle size and loading govern NO_2_ response and that maximum oxide content is not the optimum [18]. These observations are not mutually inconsistent: each experiment probes a different balance among receptor area, shell continuity, CNT percolation, interfacial barrier modulation, analyte chemistry, and operating condition. These non-universal architecture trends are summarized in Table 5.

The contradiction is therefore only apparent. A continuous shell, discrete decoration, or interpenetrating network can each be optimal when it preserves an analyte-accessible receptor surface and couples that surface strongly to the dominant current path. The appropriate design variable is not the morphology label itself, but the electrical leverage of the gas-sensitive interface under the intended analyte, temperature, humidity, and activation conditions.

Dense or fully encapsulated architectures may contain abundant oxide but often become diffusion- or transport-limited. By contrast, porous, interlaced, partially coated, or staged architectures tend to perform better because they preserve CNT continuity while exposing enough TiO_2_ surface to the target gas. This is why the highest-performing systems are often intermediate configurations rather than materials with maximum oxide fraction, maximum CNT fraction, or maximum number of components.

## 7. Sensor Benchmarking and Reporting Normalization

Table 6 lists the minimum descriptors required for meaningful cross-study comparison, and Table 7 extends this logic by linking common mechanistic claims to the controls and diagnostic evidence needed to support them.

A comprehensive review of TiO_2_–CNT gas sensors must address a basic problem: many reported performance values are not directly comparable even when they target the same analyte. The difficulty does not arise only from different materials, but also from different response definitions, baseline conventions, temperature programs, humidity levels, illumination intensities, film thicknesses, gas-flow protocols, and recovery criteria. A high response value should therefore not be interpreted as evidence of superior performance unless the operating window and reference state are explicit. A scientifically defensible hierarchy should distinguish three questions: whether a material detects the analyte, whether two sensors can be compared under equivalent conditions, and whether the device has been validated under application-relevant interference, humidity, drift and power constraints. This review treats these as progressively stronger levels of evidence rather than interchangeable demonstrations.

For chemiresistive devices, the first normalization step is to state the response equation and the sign of the resistance change. A second step is to report whether the device behaves as an oxide-dominated, CNT-dominated, or interface-dominated conductor. A third step is to distinguish sensitivity from practical usefulness. A sensor with high response at a high analyte concentration, dry atmosphere, elevated temperature, or incomplete recovery may be mechanistically interesting but less application-ready than a lower-response device that operates reproducibly under humid, interferent-rich, and low-power conditions.

Figure 3 summarizes the proposed benchmarking framework for TiO_2_–CNT gas sensors, integrating material descriptors, test conditions, sensor metrics, and validation criteria. It also highlights that quantitative comparisons are meaningful only when response definitions and operating conditions are equivalent.

### 7.1. From Plausible Mechanisms to Falsifiable Operando Tests

The most urgent mechanistic need is not another ex situ band diagram, but measurements that track the proposed interfacial process while the electrical signal is changing under a controlled gas exposure. Table 8 maps recurrent mechanistic claims to experiments that can make them falsifiable. The strongest experimental designs synchronize spectroscopy or work-function measurements with resistance, impedance, or photocurrent so that changes in chemical state and transduction can be compared on the same time axis.

These experiments are presented as methodological priorities rather than as claims that every technique is required for every sensor. Their purpose is to replace mechanism-by-analogy with mechanism-by-correlation under realistic gas exposure, while retaining the control-device logic summarized in Table 7.

### 7.2. Operationalizing the Benchmarking Framework: A Worked Example

The framework in Figure 3 can be applied as a sequence of four gates rather than as a numerical score. First, a *comparability gate* asks whether the response definition, analyte concentration, temperature, humidity, flow conditions, and activation protocol are explicit enough for comparison. Second, an *evidence gate* asks whether material identity, architecture, and reference/control devices support the claimed structure–performance relationship. Third, an *application gate* evaluates selectivity, recovery completeness, humidity robustness, drift, batch reproducibility, mechanical durability where relevant, and the total power or activation budget. Finally, a *mechanistic gate* asks whether charge-transfer, vacancy, spillover, or photoactivation claims are supported by independent diagnostic evidence rather than inferred from response magnitude alone. A study can therefore be valuable mechanistically even when it does not yet pass the application gate.

Chang et al. provide a useful example because their interlaced CNT/anatase-TiO_2_ film combines several attributes often claimed for TiO_2_–CNT sensors [16]. The study reports NO sensing over a 0.5–200 ppm test range, a 41% resistance change at 50 ppm NO at room temperature, a reported detection limit of 500 ppb, selectivity against NH_3_, NO_2_, CH_4_, and H_2_S, reversible response/recovery with UV assistance, and preservation of sensing behavior under complex mechanical deformations [16]. Under the present framework, these data clearly establish a strong Level-1 proof of concept and address important elements of selectivity, reversibility, and mechanical robustness. They also illustrate why a large response value alone should not be converted into a cross-study ranking: the result is tied to a specific NO concentration, response convention, UV-assisted recovery protocol, and flexible architecture.

For application-level benchmarking, the same dataset would be strengthened by a complete humidity dependence, the optical power and duty cycle used for recovery, long-term baseline/sensitivity drift, device-to-device and batch-to-batch statistics, and a defined bending radius/cycle-count fatigue protocol. These additional measurements would help determine whether the device can progress from a compelling flexible sensing demonstration toward the Level-3 application-relevant category in Figure 3. The worked example therefore shows how the framework is intended to be used: it records demonstrated strengths, identifies non-equivalent conditions that prevent false numerical ranking, and converts missing validation fields into specific next experiments rather than treating them as generic limitations. Table 9 summarizes this worked application of the benchmarking framework.

## 8. Sensor Performance by Target Gas

The analyte-specific literature on TiO_2_–CNT hybrids makes it clear that these materials cannot be discussed as a single sensing family with uniform behavior. Instead, the relative contribution of TiO_2_ surface chemistry, CNT percolation, interfacial barrier modulation, humidity-dependent adsorption, and external activation depends strongly on the target gas. Even within the same analyte class, apparently similar materials may respond very differently because the dominant resistance change may occur in oxide domains, mixed TiO_2_–CNT contacts, CNT junctions, or electrode interfaces. Consequently, a meaningful review must analyze sensing performance by analyte class and then identify the structural features that repeatedly govern response amplitude, kinetics, selectivity, and stability.

At the broadest level, TiO_2_–CNT gas sensing can be viewed as a coupling of receptor and transducer functions. TiO_2_ usually supplies the dominant receptor component through oxygen adsorption, vacancy-mediated activation, and analyte-sensitive surface redox chemistry. CNTs often supply the dominant transducer component through conductive percolation and amplification of local charge perturbations into measurable resistance changes. Yet this partition is rarely absolute. Defect-rich CNTs can adsorb polar gases directly, while oxide-rich regions may dominate current flow when shell continuity or particle loading is high. This distributed nature of function is the reason why analyte class matters so strongly in TiO_2_–CNT hybrids.

### 8.1. Nitrogen Oxides: NO_2_ and NO

Nitrogen oxides are among the most informative analytes for TiO_2_–CNT systems because they strongly probe the balance between oxide adsorption capacity and CNT-mediated transport at low or moderate temperature. NO_2_ is a strongly oxidizing gas and therefore highlights whether the hybrid retains sufficient TiO_2_ surface reactivity while preserving an electrically sensitive pathway through the nanotube scaffold [6,17,18,29].

Across the selected core literature, strong NO_*x*_ responses are obtained when TiO_2_ remains chemically active without electrically decoupling the CNT network, but the optimum morphology is not universally a discontinuous decoration. Interlaced films and discrete nanoparticles can be effective, whereas the ALD study of Marichy et al. showed that a thin, homogeneous 5.5 nm TiO_2_ coating outperformed discontinuous films and nanoparticle decoration at 150 °C [29]. The more defensible design rule is therefore to preserve interfacial electrical leverage: oxide coverage must be sufficient to modulate the current path, but not so thick or poorly coupled that gas-induced surface changes become screened from the CNT network.

Chang et al. reported an intrinsically flexible CNT–TiO_2_ interlaced film for NO sensing at room temperature [16]. This architecture is especially important because it deliberately separates flexible CNT segments from TiO_2_-coated segments while preserving mechanical continuity. The resulting device not only provided room-temperature response and UV-assisted recovery, but also maintained sensing performance under bending and twisting. This study shows that flexibility and sensing function need not be antagonistic when transport-rich and adsorption-rich domains are spatially organized rather than randomly mixed.

An earlier ternary example by Yun et al. is historically useful because it coupled polyaniline/MWCNT conduction with the photocatalytic action of TiO_2_ for NO sensing [58]. UV irradiation changed the chemical state of adsorbed NO-derived species and improved both sensitivity and response rate. Although the presence of polyaniline means that this is not a binary TiO_2_–CNT sensor, the study anticipated a central theme of the later literature: optical activation can alter not only carrier density in TiO_2_ but also the identity and residence time of surface reaction products, thereby changing the signal generated by the carbon-based transducer.

Fort et al. compared TiO_2_-decorated and Au-decorated SWCNT sensors for NO_2_, whereas Panzardi et al. examined TiO_2_-decorated SWCNT networks under temperature-pulsed operation [6,17]. Taken together, these studies show that surface decoration and operating protocol can both amplify oxidizing-gas response, but the contribution of TiO_2_ must be distinguished from that of alternative catalytic modifiers such as Au rather than interpreted as a single ternary mechanism.

Ito et al. extended this trend by showing that the response of CNT-based NO_2_ sensors can be increased by TiO_2_ nanoparticle decoration, with optimal performance depending strongly on particle size and oxide loading [18]. The structural implication is important: more TiO_2_ is not always better. Instead, there is a compositional and morphological optimum beyond which excess oxide accumulates without contributing proportionally to the response. This result strongly supports the broader argument that TiO_2_–CNT gas sensing is fundamentally interfacial rather than additive.

Behzadi Pour et al. provide an adjacent NO_2_-removal example using CNTs/TiO_2_ and CNTs/TiO_2_/CuO/zeolite films [59]. The work is useful here only as a boundary case: hierarchical porosity and redox-active additives can increase NO_2_ uptake, but adsorption capacity alone should not be interpreted as sensor performance unless reversibility, recovery, and electrical transduction are demonstrated.

Several closely related TiO_2_–carbon or TiO_2_-based NO_2_ studies are best treated as contextual benchmarks rather than core TiO_2_–CNT evidence. Choi et al. showed that UV activation can enable room-temperature NO_2_ response in ZnO/TiO_2_ hemitube and nanotube heterostructures; Drozdowska and Smulko showed that Au–graphene–TiO_2_ sensors can separate UV-driven TiO_2_ activation from plasmonic activation; Ling et al. showed that a small rGO fraction can bridge TiO_2_ nanotubes and enhance room-temperature NO_2_ response; and Ng et al. showed that nanometer-scale oxide-shell thickness can strongly affect NO_2_ response in TiO_2_ nanotube scaffolds [48,60,61,62]. These works support the architectural logic of TiO_2_–CNT sensing, but they should not be weighed as direct CNT-composite sensor evidence.

Patel et al. add a direct carbon/TiO_2_ NO_2_ sensor example by fabricating free-standing carbon/TiO_2_ thin films from green-synthesized carbon nanostructures obtained from onion waste and hydrothermally grown TiO_2_ [45]. The composite combined tube-like carbon structures with broccoli-flower-like TiO_2_ domains and produced a substantially larger chemiresistive response to NO_2_ than bare TiO_2_, reaching 339% at 100 ppm NO_2_ at 210 °C compared with 218% for the pristine TiO_2_ sensor [45]. Although the analyte concentration is higher than sub-ppm environmental limits, this work is valuable because it demonstrates a scalable free-standing carbon/oxide film in which the carbon phase improves electrical transduction while TiO_2_ supplies the dominant adsorption and redox-active surface. It therefore complements the CNT-specific literature by showing that self-supporting carbon/TiO_2_ geometries can simplify device fabrication and intensify NO_2_ response when carbon continuity and oxide surface exposure are retained.

The NO_*x*_ literature therefore converges on a more nuanced rule: TiO_2_ must remain electronically coupled strongly enough that adsorption changes the resistance of the hybrid, while the CNT network must remain sufficiently conductive to transduce that perturbation. Depending on thickness and contact quality, either discrete decoration or a thin conformal coating can satisfy this requirement. Architecture and interfacial continuity therefore outweigh nominal composition, but there is no universal preference for partial versus continuous oxide coverage.

Table 10 summarizes representative NO_*x*_-sensing TiO_2_–CNT systems, separating material/operation descriptors from the critical interpretation of advantages and limitations.

### 8.2. Ammonia

Ammonia is a major benchmark analyte for low-temperature sensing because it is industrially relevant, humidity-sensitive, and mechanistically complex. In TiO_2_–CNT hybrids, NH_3_ sensing lies at the intersection of acid-base chemistry, defect-mediated adsorption, and barrier modulation across mixed conductive pathways. This makes ammonia especially useful for distinguishing genuinely interfacial sensing from simple physical mixing.

Direct TiO_2_–CNT evidence is provided by Kaushik et al., who deposited 5, 10 and 20 nm TiO_2_ films on MWCNT networks by ALD and evaluated NH_3_ sensing at room temperature [30]. The 5 nm coating gave the highest response, and introducing a carboxyl plasma-polymer layer before TiO_2_ deposition improved coating uniformity and response relative to both pristine CNTs and CNT/TiO_2_ structures without the interlayer. This experiment is particularly valuable because it separates two effects that are often conflated: chemical functionalization can improve oxide nucleation and interfacial coupling, yet increasing oxide thickness can simultaneously introduce CNT disorder and reduce the electrical leverage of the nanotube network. The optimum is therefore a thin, well-coupled receptor layer rather than the maximum attainable oxide loading.

Hwang et al. provide a useful oxide-only benchmark for room-temperature NH_3_ detection because their Au nanoparticle-decorated TiO_2_ nanosheets isolate two variables that are also central to TiO_2_–CNT sensors: surface oxygen defects and catalytic spillover [49]. The authors prepared monolayer TiO_2_ nanosheets by flux growth and chemical exfoliation, followed by hydrothermal Au decoration, obtaining a response of approximately 2.8 toward 20 ppm NH_3_ at room temperature; this response was markedly higher than that of pristine TiO_2_ nanosheets and was attributed to the increase in oxygen-vacancy-related surface states and Au-assisted activation of adsorbed oxygen species [49]. Although CNTs were not present in this sensor, the study clarifies why noble-metal decoration and low-dimensional TiO_2_ morphologies are powerful design levers for NH_3_ sensing. In TiO_2_–CNT hybrids, an analogous role can be envisioned when CNTs preserve low-resistance transport while defective or metal-decorated TiO_2_ domains provide NH_3_ adsorption, oxygen activation, and interfacial barrier modulation.

Seekaew et al. demonstrated that TiO_2_-containing carbon-based hybrids can achieve highly selective NH_3_ detection at room temperature [22]. The significance of this result is not merely the magnitude of the response, but the fact that high selectivity can be maintained under mild conditions where many bare oxides remain sluggish or poorly reversible. In this class of materials, TiO_2_ contributes polar adsorption sites and oxygen-related chemistry, while the carbon framework preserves transport and enables operation without substantial external heating.

Bakos et al. approached the problem through deposition-controlled oxide growth on MWCNT scaffolds [15]. More specifically, their ALD-derived CNT–TiO_2_–ZnO and CNT–ZnO–TiO_2_ heterostructures showed that NH_3_ response is not governed by oxide loading alone but by the conductivity contrast and surface chemistry of the terminal shell. The configuration with TiO_2_ as the outer layer produced substantially larger NH_3_ responses than the corresponding ZnO-terminated structure, even though both shared the same carbon core and comparable multilayer character [15]. This observation is especially important because it shifts the design rule from a simplistic “more oxide is better” logic to a more precise interfacial principle: for reducing gases such as NH_3_, the shell that directly contacts the analyte must combine sufficient adsorption affinity with a resistance level high enough for the adsorption event to induce a meaningful transduction signal.

More complex architectures, such as the Sn-TiO_2_/rGO/CNT systems of Seekaew et al. and the TiO_2_–SnO_2_–MWCNT–MOF-related configurations of Wong et al., further indicate that ammonia sensing can benefit from orthogonal amplification strategies, including oxide adsorption, carbon transport, porous preconcentration, and heterojunction-driven barrier modulation [22,64]. Even when these systems move beyond a strict binary TiO_2_–CNT composite, they remain highly instructive because they reveal how TiO_2_–CNT principles can be embedded into broader hybrid sensing platforms.

Machín et al. provide a useful CNT-centered benchmark for this discussion because their SWCNT-based NH_3_ sensors isolate the effect of noble-metal and MoS_2_ sensitization on a carbon transduction network [65]. Vertically grown SWCNTs were modified with Au nanoparticles either by sputtering or chemical deposition, and an additional MoS_2_–AuNP–SWCNT device was prepared by incorporating exfoliated MoS_2_ nanosheets [65]. The sputtered AuNP–SWCNT sensor showed faster NH_3_ response than the chemically impregnated analogue, with response/recovery times of about 12/52 s at 140 °C for sub-ppm to 1 ppm NH_3_ exposure; the MoS_2_–AuNP–SWCNT configuration further improved recovery behavior, including at room temperature [65]. For TiO_2_–CNT ammonia sensors, this work is best used as a mechanistic analogue rather than a compositional match: it demonstrates that the distribution and deposition route of catalytic nanoparticles can be as important as their nominal loading, and that adding a layered semiconductor can accelerate recovery by introducing additional adsorption and charge-transfer pathways. This reinforces a key design principle for NH_3_ sensing: low-temperature reversibility often requires a carefully engineered carbon network plus catalytic or semiconducting modifiers, not only a high density of adsorption sites.

Orlando et al. provide a practical SWCNT benchmark for both NO_2_ and NH_3_ by showing that even the dispersing agent used during film fabrication can alter chemiresistive performance [66]. Their spray-deposited SWCNT sensors, prepared with carboxymethyl cellulose or sodium dodecyl sulfate surfactants, were selective toward NO_2_ and NH_3_ relative to ethanol and CO, and the best NO_2_ performance was obtained for 10 deposited layers at 150 °C and 40% relative humidity, with responses of 78.6% for the CMC-based sensor and 62.5% for the SDS-based sensor toward 2 ppm NO_2_ [66]. For TiO_2_–CNT hybrids, this study is not a TiO_2_ example but it is methodologically important: CNT dispersion chemistry, residual surfactant, film thickness, humidity, and operating temperature can be decisive variables, so differences attributed to oxide decoration may sometimes partly originate from the carbon-network preparation route.

The NH_3_-focused review by Norizan et al. is useful here as a broader CNT-centered context because it summarizes why CNT-based ammonia sensors can operate at low temperature while metal-oxide sensors often require elevated temperatures [67]. Their discussion emphasizes that NH_3_ detection in CNT films is governed by adsorption-induced changes in resistance, conductivity, work function, or permittivity, but that pristine CNTs often suffer from limited selectivity, slow regeneration, and weak interaction with gas molecules [67]. For TiO_2_–CNT hybrids, this supports the rationale for using TiO_2_ or other semiconducting modifiers as chemically active receptor domains while retaining CNTs as low-resistance transduction pathways. The article is therefore best integrated as a contextual review source, not as a primary TiO_2_–CNT performance benchmark.

Humidity, however, remains a decisive complication. NH_3_ and H_2_O compete for adsorption sites, and water can also facilitate protonic transport or alter the surface chemistry of both TiO_2_ and functionalized CNTs. A substantial fraction of the apparent disagreement in NH_3_ sensing literature likely reflects differences in humidity control rather than intrinsic superiority of one hybrid over another. For that reason, room-temperature NH_3_ sensing should be interpreted only alongside humidity conditions and recovery protocol.

### 8.3. Hydrogen and Hydrogen-Related Reducing Gases

Hydrogen remains one of the clearest cases where TiO_2_–CNT hybridization yields a practical gain. Pure TiO_2_ often suffers from high resistance and weak room-temperature response, whereas CNT incorporation can reduce the transport bottleneck while preserving access to active oxide domains. The crucial point, however, is that the gain does not arise from composition alone but from architecture.

The mechanism was examined directly in the Pt/TiO_2_/CNT system of Santangelo et al. [28]. The sensor operated near room temperature in helium over a very broad H_2_ concentration range, while systematic variation of CNT loading showed a non-monotonic response with an optimum around 34 wt% CNT. The authors related this optimum to changes in TiO_2_ stoichiometry, nanoparticle agglomeration and TiO_2_–CNT contact and proposed that hydrogen-related electron transfer through the oxide quenches the conductance of the CNT network. This study remains unusually valuable because the sensing mechanism was tested against deliberate compositional variation rather than inferred from a single optimized sample.

Abdulhameed and Mahnashi provided one of the most informative examples by fabricating a H_2_ sensor using two-stage dielectrophoresis [14]. A conductive CNT network was assembled first, and TiO_2_ nanoparticles were deposited only afterward using a different DEP condition. This staged method yielded substantially better H_2_ response and much lower resistance than a one-step mixed route. Mechanistically, the result is compelling because it demonstrates that TiO_2_ should be placed as an interfacial modifier rather than randomly dispersed in the conductive phase.

Mohd Chachuli et al. provide a more direct H_2_-sensing example in a TiO_2_/MWCNT/B_2_O_3_ thick-film platform prepared by screen printing and tested against 100–1000 ppm H_2_ at 100–300 °C [68]. Their comparison of linseed-oil and ethyl-cellulose binders is useful because it shows that apparently secondary processing variables can alter conductivity, recovery, and sensitivity even when the nominal TiO_2_/MWCNT/B_2_O_3_ composition is maintained. The ethyl-cellulose-derived sensor showed better sensitivity, whereas the linseed-oil-derived sensor showed better conductivity and recovery behavior [68]. This finding reinforces a practical design rule for TiO_2_–CNT gas sensors: paste formulation, residual carbon, binder burnout, and film cohesion should be treated as part of the sensing architecture rather than as purely fabrication details. As in other analyte classes, the literature repeatedly suggests that network topology is more decisive than nominal composition. CNTs must remain electronically connected after oxide integration if the hybrid is to outperform its individual constituents.

Hydrogen-related design principles also extend naturally to visible-light-assisted formaldehyde sensing. In the Pd/TiO_2_ system built on carbon nanotube interdigital electrodes, Liu et al. demonstrated trace HCHO detection at room temperature under visible light [25]. Although formaldehyde rather than H_2_ is the target analyte, the logic is the same: catalytic activation at the oxide surface, efficient low-temperature transduction, and a carbon-based conductive framework that allows weak gas-induced perturbations to be collected efficiently. These studies suggest that TiO_2_–CNT architectures are especially attractive whenever room-temperature detection of reducing gases would otherwise be limited by poor oxide conductivity.

A related but non-sensing hydrogen study by Farah et al. is useful for defining the upper mechanistic potential of TiO_2_–CNT–metal interfaces [44]. They reported that TiO_2_/CNT increased photocatalytic H_2_ production from water/methanol relative to TiO_2_ alone, and that introducing Au-decorated CNTs further amplified production because Au and CNTs acted synergistically rather than additively. The same system was integrated as a CNT-retained packed bed in a microfluidic reactor, where TiO_2_/AuCNT produced more H_2_ than TiO_2_/CNT under continuous flow. For the present review, the value of this work is conceptual: it demonstrates that TiO_2_–CNT–Au junctions can combine charge separation, plasmonic sensitization, and robust nanoscale immobilization, all of which are also relevant to low-temperature hydrogen-related gas sensing [44].

### 8.4. Hydrogen Sulfide and Sulfur-Containing Analytes

H_2_S is less represented within the selected TiO_2_–CNT corpus than NO_*x*_, NH_3_, or VOC-related analytes, yet it remains strategically important because it exposes both the opportunities and the limits of TiO_2_-centered sensing under low-temperature conditions. H_2_S is a chemically aggressive reducing gas whose interaction with oxide surfaces can lead to strong signal generation, but also to selectivity challenges when competing reducing gases are present.

Chen et al. introduced a distinct perspective by using TiO_2_/MWCNT-related functional layers in a fiber-optic surface plasmon resonance sensor for H_2_S detection [21]. This contribution is valuable because it expands the discussion beyond purely chemiresistive transduction. In their system, the hybrid layer modulates optical rather than merely resistive properties, demonstrating that TiO_2_–CNT composites can operate as multifunctional interfaces in which adsorption affects dielectric and plasmonic behavior.

The importance of this result is broader than the single device. One of the persistent limitations of chemiresistive gas sensors is insufficient selectivity. Optical and optoelectronic readout strategies may offer a richer information channel, and TiO_2_–CNT hybrids are especially well suited to such strategies because they combine surface chemistry, electrical conductivity, refractive response, and interfacial defect states. For a comprehensive review, this is a reminder that TiO_2_–CNT hybrids should not be constrained conceptually to resistance-only sensing.

### 8.5. Acetone, Formaldehyde, Toluene, and Other VOCs

Volatile organic compounds constitute the most diverse analyte class in the TiO_2_–CNT literature. Here, sensitivity alone is not enough. Selectivity, humidity tolerance, low-temperature operation, and, in many cases, diagnostic relevance become central. This makes the VOC literature particularly important for moving TiO_2_–CNT hybrids from proof-of-concept materials to application-oriented sensing systems.

The broader carbon-nanomaterial VOC-sensing review by Nath et al. is useful here because it frames CNTs, graphene derivatives, carbon dots, and related carbon phases as tunable VOC transducers whose response depends on exposed surface area, π-electron interactions, charge transfer, and functional groups [69]. The review emphasizes that VOC detection with carbon nanostructures is governed by adsorption, interaction at active sites, and desorption, with resistance changes arising from charge-transfer or mobility modulation [69]. For the present TiO_2_–CNT review, this provides a general interpretive bridge: TiO_2_ introduces oxide-mediated adsorption and oxygen chemistry, whereas CNTs contribute carbon-surface affinity and percolative transduction, making VOC selectivity especially dependent on interface chemistry rather than on either component alone.

The hydrothermal TiO_2_/MWCNT study of Everhart et al. is also relevant to VOC-oriented discussion because acetaldehyde is a representative gas-phase indoor pollutant: the optimum 1 wt% MWCNT composite reached substantially faster UV photocatalytic degradation than pristine TiO_2_, whereas excessive CNT content penalized performance through agglomeration and light-shielding effects [43].

Hwang et al. [7] reported a breath-acetone sensor based on oxidized single-walled carbon nanotubes functionalized with TiO_2_. In their terminology, SWCNT@TiO_2_ denotes a hybrid architecture in which TiO_2_ is anchored, deposited, or grown on the surface of single-walled carbon nanotubes; thus, the “@” symbol indicates an interfacial configuration rather than a simple physical mixture. The key contribution of this work is not only that acetone can be detected by a SWCNT@TiO_2_ chemiresistor, but that the authors explicitly recognized and engineered around the system’s strongest limitation: water vapor. Because the initial device showed strong cross-sensitivity to humidity, a custom dehumidifier was introduced upstream of the sensor. This is highly significant from an editorial standpoint because it shows that, in realistic breath-analysis settings, humidity management is not ancillary but part of the sensing architecture itself.

Formaldehyde sensing provides another important example of how TiO_2_–CNT platforms can be pushed toward low-temperature or trace-gas operation. Liu et al. used visible-light-assisted Pd/TiO_2_ with CNT electrodes to enable trace HCHO detection at room temperature [25]. The paper is relevant not only because of its application target, but because it demonstrates how catalytic activation, light assistance, and carbon-based electrodes can be combined to overcome the weak signal problem associated with trace-gas sensing. Tan et al. further showed that multitype carbon doping and oxygen vacancies in TiO_2_–CNT composites can profoundly alter photocatalytic formaldehyde removal by changing interfacial bonding, light response, and the direction of electron transfer [35]. Although that work is more photocatalytic than sensor-centric, it is highly valuable for the mechanistic sections of this review because it reveals how TiO_2_–CNT interfaces may behave when vacancies and interfacial C–O–Ti bonding are intentionally engineered. A related recent study by Akhter et al. synthesized TiO_2_/CNT nanocomposites by a hydrothermal route and reported more than 85% visible-light methylene blue degradation together with five-cycle stability, attributing the improvement to better charge separation, suppression of TiO_2_ agglomeration, and Ti–O–C/Ti–C interfacial connectivity [70]. Although focused on wastewater remediation rather than gas sensing, the paper reinforces a central mechanistic theme of this review: CNT incorporation can simultaneously increase accessible surface area, facilitate electron extraction, and stabilize photoinduced carriers, all of which are directly relevant to light-assisted TiO_2_–CNT sensing.

Greenstein et al. provide a useful adjacent benchmark from reactive water filtration, where electrospun carbon/TiO_2_ nanofiber composites were carbonized to obtain mechanically robust, flow-through photocatalytic filters for emerging organic contaminants [71]. Although the carbon phase is not CNT-based, the work is relevant because it isolates several architecture-level variables that also matter in TiO_2_–CNT sensors: embedded TiO_2_ loading, surface-accessible Ti sites, carbon-supported charge management, and the balance between photocatalytic activity and mechanical stability. Their optimized C/TiO_2_ filters removed diverse micropollutants under UV-assisted filtration, showing that carbon–TiO_2_ interfaces can be engineered as practical reactive platforms rather than only as powder photocatalysts [71].

Hastuti et al. add a particularly relevant membrane-based benchmark because they moved TiO_2_/CNT from a powder or thin-film photocatalyst into a mechanically usable PAN nanofiber platform for continuous cross-flow operation [72]. Their PAN/TiO_2_/CNT and PAN/TiO_2_/CNT-PVA electrospun membranes retained anatase TiO_2_ and showed XPS/Raman evidence of Ti–O–C interfacial bonding, with CNTs acting as electron sinks that delay electron–hole recombination and extend visible-light activity under a low-power LED source [72]. Under cross-flow filtration, PAN/TiO_2_/CNT maintained 100% methylene-blue rejection during the first 8 h, while reusability tests showed that the membrane preserved more than 80% rejection after five 16 h cycles; the PVA-coated version increased hydrophilicity and mechanical resistance, although stronger dye adsorption complicated complete flux recovery after repeated cycles [72]. For TiO_2_–CNT gas sensors, the direct relevance is architectural rather than analyte-specific: immobilizing the hybrid in a polymer nanofiber scaffold can preserve interfacial charge separation, reduce catalyst leaching, improve mechanical durability, and maintain accessible pathways for mass transport. This makes the study a useful analogue for future flexible, filter-integrated, or flow-through sensing layers in which photocatalytic self-cleaning, humidity control, and gas access must be balanced within the same porous architecture.

Li et al. provide a closely related TiO_2_–CNT/polymer-nanofiber analogue by loading acid-treated CNTs and hydrothermally generated TiO_2_ nanoparticles onto electrospun PMMA nanofibers [73]. Although the target application was dye photodegradation rather than gas detection, the study is valuable for the present review because it combines three sensor-relevant design elements in a single architecture: a CNT phase for electron extraction, an anatase TiO_2_ phase for photocatalytic redox activity, and a recyclable porous nanofiber scaffold for mass transport and mechanical handling. The TiO_2_/CNTs/PMMA composite reached a BET surface area of 83.2 m^2^ g^−1^, fully degraded 10 mg L^−1^ methyl orange within 40 min under UV irradiation, and retained high activity over ten consecutive cycles [73]. Importantly, the acid treatment of CNTs improved compatibility with TiO_2_ and PMMA, promoted more uniform TiO_2_ anchoring, and strengthened intimate TiO_2_–CNT contact, thereby improving charge separation relative to TiO_2_/PMMA and untreated-CNT controls [73]. For gas-sensing materials, this work supports the idea that polymer-supported TiO_2_–CNT mats can serve as mechanically robust, recoverable, and highly accessible platforms in which photocatalytic self-cleaning and photoassisted recovery could be coupled to chemiresistive transduction.

Lian et al. extend this immobilization logic to a microstructured platform by embedding MWCNT/TiO_2_ nanocomposites within PDMS microparticles produced through needle-based microfluidics [74]. The work is not a gas-sensor report, but it is highly relevant as an architectural analogue because it combines sorption by a polymer matrix with photocatalytic degradation by a TiO_2_–CNT phase, thereby addressing two recurrent limitations of TiO_2_–CNT systems: insufficient analyte preconcentration and difficult recovery of nanoscale photocatalysts. The MWCNT/TiO_2_ nanocomposite was prepared by a surfactant-wrapping sol–gel route that deposited anatase TiO_2_ uniformly on acid-treated MWCNTs, while the PDMS microparticles enabled facile handling and reuse [74]. The optimized PDMS–MWCNT/TiO_2_ microparticles reached 85% Rhodamine B removal and retained about 70% efficiency after three reuse cycles [74]. For TiO_2_–CNT gas sensors, this study supports a broader design principle: polymeric or elastomeric hosts can be used not only as passive supports, but also as analyte-concentrating reservoirs coupled to CNT-assisted TiO_2_ photoactivity, which may be useful for self-cleaning, preconcentrating, or regenerable sensing layers.

Horváth et al. provide a second water-treatment benchmark that is even closer to the TiO_2_–CNT gas-sensing problem because their freestanding filter was built directly from TiO_2_ nanowires interwoven with CNTs and operated only with sunlight [75]. The composite retained microbial pathogens by nanoporous filtration while UV-excited TiO_2_ generated reactive oxygen species, and the CNT phase added two functions that are highly transferable to sensor design: electron extraction from photoexcited TiO_2_, which suppresses recombination, and broadband solar absorption, which produced a photothermal pasteurization channel [75]. Their prototype reached temperatures near 50 °C under sunlight, generated ROS verified by TEMPOL/TEMPONE ESR analysis, degraded methyl orange more efficiently than a P25 reference under UVA illumination, reduced selected micropollutants after repeated passages, and inactivated *E. coli* rapidly when bacteria were trapped at the filter surface [75]. For the present review, this work is treated as a contextual architectural analogue rather than as gas-sensor evidence. It demonstrates that a porous TiO_2_–CNT platform can combine mechanical capture, photocatalytic oxidation, and photothermal activation in a self-standing architecture. These functions suggest testable design options for analyte-accessible, light-regenerated sensing layers, but they do not establish gas-sensing response, selectivity, recovery, or power requirements.

Hossen et al. recently systematized this broader cross-domain logic in a comprehensive review of carbon-based nanomaterial-modified TiO_2_ nanotubes for photo-driven energy and environmental applications [76]. Their analysis is useful for the present gas-sensing review because it frames TNT/CNM hybrids as platforms in which one-dimensional TiO_2_ morphology, high surface area, oriented electron transport, and tunable nanotube geometry can be coupled with the photosensitization, band-gap narrowing, co-catalytic flexibility, porosity, and active-site dispersion provided by carbon-based phases [76]. Although their scope emphasizes water splitting, CO_2_ utilization, pollutant removal and detection, photovoltaic cells, energy storage, and photocathodic protection, the mechanistic framework is useful here only for hypothesis generation. Carbon-assisted light harvesting, charge separation, adsorption/redox activity, and conductive transport are plausible functions to test in TiO_2_–CNT gas sensors, but their magnitude and even their relevance to the measured sensor current must be verified under gas exposure rather than transferred directly from those applications. The review also notes that emerging carbon modifiers such as MXenes and MOF-derived carbons remain at an early stage for TNT composites, and that most high-performing CNM–TNT systems are still confined to laboratory-scale demonstrations [76]. This observation reinforces a key challenge for TiO_2_–CNT gas sensors: future progress will depend not only on higher response values, but also on scalable fabrication, interface reproducibility, long-term stability, and integration of carbon-assisted photoactivation into device architectures.

Li et al. also illustrate the value of three-dimensional TiO_2_–CNT architectures from an energy-storage perspective by electrodepositing TiO_2_ networks directly onto CNT films and converting them into nanobranched rutile-rich TiO_2_–CNT electrodes [77]. This work is not a gas-sensing study, but it is useful for this review because it separates the structural and transport functions of the CNT network: CNTs act simultaneously as conductive scaffolds and current collectors, while the TiO_2_ nanobranches provide a high-surface-area oxide interface. The optimized electrode showed Ti–O–C bonding signatures, a surface area up to 324 m^2^ g^−1^ after 900 °C annealing, a specific capacitance of 345.7 F g^−1^ at 1.0 A g^−1^, an energy density of 82.5 Wh kg^−1^, and 93.3% capacitance retention after 10,000 cycles [77]. For TiO_2_–CNT sensors, the transferable lesson is that vertically connected or nanobranched oxide networks can shorten ion or molecule diffusion paths while preserving electronic transport through the CNT backbone. Such architectures may be especially useful for low-power sensing layers that must combine high interfacial area, robust electrical pathways, and long-term cycling stability under repeated adsorption, desorption, and photoactivation events.

Li et al. further broaden the relevance of TiO_2_–CNT interfaces by using sulfated TiO_2_ supported on CNTs, ${\mathrm{SO}}_{4}^{2-}$/TiO_2_–CNT, as a bifunctional catalyst for low-energy CO_2_ desorption during MEA regeneration [78]. Although this application belongs to carbon capture rather than gas detection, it provides a particularly useful mechanistic analogue because the same hybrid platform couples acid-site chemistry on TiO_2_ with charge-transfer functionality from CNTs. The optimized STC-1.0 catalyst increased the CO_2_ desorption amount by 82.3% and reduced the relative heat duty by 46% compared with the uncatalyzed MEA–CO_2_ solution, while maintaining stable performance over 15 absorption–desorption cycles [78]. The authors attributed this behavior to synergistic proton and charge transfer: sulfated TiO_2_ promotes carbamate decomposition through acid sites, whereas CNTs facilitate bicarbonate decomposition through delocalized charge transfer [78]. For TiO_2_–CNT gas sensors, the value of this work is conceptual: it shows that CNTs can be paired with chemically functionalized TiO_2_ not only to improve conductivity, but to create bifunctional interfaces in which adsorption, proton transfer, and electron transfer are tuned simultaneously.

Because the manuscript is already extensive, other cross-domain TiO_2_–CNT studies are best used only as compact mechanistic support rather than as separate discussion blocks. For instance, Cu- and Fe-modified V_2_O_5_/TiO_2_–CNT catalysts for NH_3_-SCR of NO_*x*_ show that CNT-containing TiO_2_ supports can improve low-temperature NO_*x*_ conversion and resistance to interfering SO_2_/H_2_O, but these studies should be cited mainly as evidence of robust NO_*x*_ surface redox chemistry rather than as gas-sensor precedents [79,80]. Similarly, microwave-assisted CNTs/TiO_2_ photocatalysis demonstrates that CNTs can act simultaneously as microwave absorbers and electron-conducting pathways, promoting charge separation, oxygen activation, and radical generation under an external field [81]. Finally, the stabilization of single atoms on defect-engineered CNT and TiO_2_ supports underscores a more general interfacial lesson: vacancy-mediated charge transfer can regulate catalytic selectivity and stability, although this contribution remains too distant from gas sensing to warrant extended treatment in the main text [82].

Photocatalytic TiO_2_–carbon studies are useful only when they clarify carrier-separation or activation principles. In this restricted sense, N-doped TiO_2_/g-C_3_N_4_ architectures and TiO_2_/CNT/Pd–Cu systems support the idea that visible-light harvesting, electron-sink sites, and carbon-mediated transport can suppress recombination and alter surface redox kinetics, but these studies remain supporting evidence rather than direct gas-sensor benchmarks [83,84].

Toluene sensing was explored by Seekaew et al. using TiO_2_ nanoparticle-decorated three-dimensional graphene-CNT nanostructures [23]. Even though graphene is also present, the work remains highly relevant because it demonstrates a room-temperature aromatic VOC sensor in which TiO_2_ decoration substantially improves sensitivity and selectivity relative to carbon-only controls. The proposed mechanism, involving Schottky-like interactions between metallic carbon nanostructures and semiconducting TiO_2_, underscores the broader point that TiO_2_/carbon hybrids frequently operate through barrier modulation rather than simple adsorption alone.

Photoactivated VOC sensing adds another layer of design complexity. Li et al. prepared TiO_2_ nanotube/CNT/ZnS quantum-dot composites and showed that UV activation enables low-temperature ethanol sensing with improved sensitivity, selectivity, and stability relative to higher-temperature TiO_2_ operation [19]. This study is representative of a broader trend in the field: when VOC adsorption is weak at room temperature, photoactivation can shift the system into a more favorable transduction regime without the energetic penalty of classical thermal heaters.

The GO/TiO_2_ chemiresistor reported by Lee et al. is included as a limited carbon–oxide analogue because it combines room-temperature gas response with UV-induced modification of the carbon phase [85]. Its main implication for TiO_2_–CNT systems is that photoactivation may change not only TiO_2_ surface chemistry but also the electronic state of the carbon network.

Finally, Shooshtari and Salehi moved beyond single-analyte response by using aligned CNT–TiO_2_ hybrid nanostructures in an electronic-nose configuration [8]. Their strategy is particularly forward-looking because it shows that a single hybrid sensing material can generate distinct response patterns when combined with different electrode materials and signal-processing protocols. The contribution is therefore not limited to a larger single-analyte response; it demonstrates a more information-rich analytical platform. This is one of the clearest indications that TiO_2_–CNT hybrids are well suited to array-based VOC discrimination rather than only single-gas/single-sensor operation.

### 8.6. Humidity and Oxygen-Related Sensing

Humidity deserves separate treatment because it is simultaneously an interference source, a mechanistic probe, and an analyte of practical interest. Estefani et al. recently demonstrated impedimetric relative-humidity sensing with TiO_2_ quantum dots integrated with acid-treated MWCNTs, reporting a six-order impedance decrease from 5 to 85% RH and frequency-dependent operation in which low frequencies enhanced sensitivity, whereas 15 kHz improved baseline stability and recovery [37]. For this review, the main value of that contribution is mechanistic: it confirms that hydroxylated TiO_2_ domains, CNT-assisted conduction, adsorbed water layers, and proton-transport pathways can dominate the electrical response when the TiO_2_–CNT interfacial area is high [37].

Lee et al. provide a useful mechanistic complement by isolating humidity response according to CNT electronic type rather than treating CNTs as a generic conductive additive [86]. Using highly purified 99% semiconducting and metallic single-walled CNT network films contacted with Pd electrodes, they showed opposite resistance trends as relative humidity increased: semiconducting CNT devices decreased in resistance, whereas metallic CNT devices increased in resistance [86]. The distinction was attributed to different dominant transduction pathways. In metallic CNT networks, adsorbed H_2_O donates electrons and compensates the oxygen-induced hole doping of the CNT channel, thereby reducing carrier density; in semiconducting CNT networks with Pd contacts, however, moisture modifies the Pd/PdH_*x*_ contact and lowers the effective hole Schottky barrier, producing a much larger response [86]. Control devices with Cu electrodes confirmed that the electrode/CNT contact can dominate the humidity response polarity and magnitude. For TiO_2_–CNT gas sensors, this result is important because humidity effects cannot be assigned only to water adsorption on TiO_2_ or functional groups on CNT walls; the metallic/semiconducting fraction of the CNT network and the electrode material can also determine baseline drift, response sign, and apparent analyte sensitivity.

The significance of humidity extends far beyond humidity sensors themselves. In many TiO_2_–CNT gas sensors, water changes the baseline, modifies oxygen adsorption, competes with target analytes, and may even introduce protonic transport pathways. Kumar et al. are particularly relevant in this regard because their MWCNT/TiO_2_ nanocomposite explicitly linked LPG sensing with humidity sensitivity and supported the interpretation with DFT calculations [26]. Their results reinforce the conclusion that real-device behavior often depends on coupled analyte/water effects rather than idealized dry-gas chemistry alone.

Oxygen-related sensing, although less common in the TiO_2_–CNT gas-sensing literature, provides a revealing example of how far hybrid systems can be extended beyond classical oxide chemisensors. Wettstein et al. [24] developed a dye-sensitized SWCNT–TiO_2_ chemiresistor capable of O_2_ detection under visible-light illumination at room temperature. Inspired by dye-sensitized solar-cell principles, the device relies on photoinduced charge transfer in a SWCNT–TiO_2_ hybrid bearing a molecular photosensitizer. This contribution is important not only because it expands the analyte space, but also because it shows that concepts from light harvesting and molecular sensitization can be translated into robust, low-power sensing schemes based on carbon–oxide interfacial functionality. These activation pathways are interpreted within the multiscale transduction framework of Figure 1; illumination and water content are therefore treated as operational variables capable of changing the dominant sensing route rather than as secondary experimental details.

## 9. Cross-Analyte Design Matrix

Table 11 converts the analyte-by-analyte discussion into a compact design matrix, making explicit which architecture, activation mode, and risk factor should dominate interpretation for each gas class.

The analyte-by-analyte discussion shows that TiO_2_–CNT hybrids should not be optimized through a universal formulation. Each gas probes a different balance between adsorption strength, charge transfer, recovery, humidity tolerance, and transport continuity. A useful synthesis is therefore to connect target-gas chemistry with architecture selection and benchmarking priorities.

This matrix also clarifies why the most convincing future papers will be those that report negative results and boundary conditions. Showing when TiO_2_ loading becomes excessive, when CNT oxidation becomes damaging, or when illumination produces drift is as valuable as reporting the highest response, because these limits define the true design space of the hybrid interface.

## 10. Activation Strategies and Operating Conditions

Humidity and activation mode reshape the operating window of TiO_2_–CNT sensors and must therefore be controlled before performance claims are compared. Their role is incorporated explicitly into the benchmarking framework in Figure 3 and the design roadmap in Figure 4.

One of the main reasons TiO_2_–CNT hybrids are so versatile is that their sensing behavior can be modulated substantially by the activation mode rather than by sensing composition alone [1,2,3,5]. Some systems operate chemiresistively at room temperature, others rely on moderate thermal activation, and still others exploit UV or visible-light irradiation to modify oxygen coverage, enhance carrier generation, or accelerate recovery [16,19,24,25]. This operational flexibility is a major advantage, but it also complicates cross-study comparison because materials with similar nominal compositions may exhibit markedly different responses under different activation conditions [8,17,27].

### 10.1. Photoactivation and Sensitization

Photoactivation is among the most powerful strategies in this field. In TiO_2_-containing systems, UV irradiation can generate electron–hole pairs, alter the ionosorbed oxygen population, and accelerate desorption and recovery. When CNTs are coupled to TiO_2_, they may act as sinks or transport channels for photogenerated electrons, reducing recombination and improving interfacial transduction [13,19,25]. In this context, CNTs do not merely lower baseline resistance; they also reshape the photophysical dynamics of the oxide.

The broader visible-light TiO_2_ literature reviewed by Liao et al. is useful for framing why carbon modification should be treated as an optical and interfacial design variable, not only as an electrical additive [87]. Their review emphasizes that visible-light activation of TiO_2_ can be achieved through metal and non-metal doping, plasmonic noble metals, semiconductor coupling, and carbonaceous modifiers such as CNTs and graphene; these strategies extend absorption into the visible region, suppress electron–hole recombination, and promote reactive oxygen species or surface oxygen populations. For TiO_2_–CNT gas sensors, the same principles translate into three design levers: band-gap or defect-state engineering to access longer wavelengths, carbon-mediated extraction of photoelectrons, and control of ROS/ionosorbed oxygen populations at the gas-accessible surface [87].

This interpretation is supported by the N-CNT/TiO_2_ thin films of Belkhanchi et al., where N-CNT addition produced a substantial decrease in the apparent optical gap and a concurrent increase in conductivity, consistent with enhanced visible-region absorption and more effective charge transport through the carbon-modified oxide network [88]. Their later rutile nanofilm work strengthens this argument by combining XRD, SEM/AFM/TEM, optical transmittance, and sheet-resistance measurements to show that annealing-controlled phase evolution and N-CNT loading jointly determine roughness, porosity, band-gap narrowing, and conductive-pathway formation in N-CNT/TiO_2_ films [89].

The ZnO/TiO_2_ work of Choi et al. adds an instructive analogue for NO_2_ sensing: the device required UV illumination for room-temperature operation, and the authors linked the response enhancement to photoadsorbed oxygen, carrier generation, and barrier modulation at the oxide heterointerface [60]. This reinforces a general design principle for TiO_2_–CNT sensors, namely that optical activation is most effective when it is coupled to an architecture capable of converting local surface photochemistry into a measurable network-resistance change.

The Au–graphene–TiO_2_ sensor of Drozdowska and Smulko further shows that different wavelengths can activate different interfacial subregions within a carbon–oxide–metal network [48]. For TiO_2_–CNT sensors, the important lesson is not the exact graphene architecture, but the need to report wavelength, intensity, and recovery protocol because UV activation, plasmonic activation, and CNT-mediated transport can contribute differently to the measured signal.

This behavior is illustrated most directly by TiO_2_/CNT-based sensing platforms that use UV-assisted recovery, photoactivated VOC response, visible-light-assisted Pd/TiO_2_ sensing, or dye-sensitized O_2_ detection [16,19,24,25]. The common design principle is that light should be treated as an active part of the sensing mechanism, not as a generic auxiliary condition.

Peón-Díaz et al. further clarify why CNT pretreatment must be treated as a primary design variable rather than a preparative detail [36]. By varying the oxidative functionalization time of CNTs before CVD deposition of TiO_2_, they showed that the extent of nitrogen- and oxygen-containing surface groups controls TiO_2_ homogeneity, film stability, optical band gap, and photoelectrochemical response; the 90 min oxidation treatment produced the most homogeneous TiO_2_ overlayer and the highest photocurrent, approximately 160 μA cm^−2^ at 0 V vs. Ag/AgCl [36]. For gas sensing, the transferable lesson is direct: CNT oxidation can improve TiO_2_ anchoring and interfacial charge extraction, but it must be optimized because under-functionalized CNTs lead to poor oxide coverage, whereas over-oxidation can damage the conductive scaffold that enables chemiresistive transduction.

Even though these studies did not measure gas response, they strengthen an important mechanistic point for this review: photoactivation in TiO_2_–CNT hybrids is highly sensitive to how the carbon phase and secondary catalytic components modify light harvesting, carrier separation, and interfacial recombination pathways. These effects can become non-monotonic once excess carbon begins to perturb transparency, uniformity, or active-site accessibility [84,90,91,92]. Taken together, these studies indicate that light activation should not be regarded only as an auxiliary tool for accelerating recovery; rather, it can become an integral part of the sensing mechanism by modulating carrier generation, interfacial charge transfer, surface redox chemistry, and desorption kinetics. The photochemical contribution can be summarized schematically as

(7) $$\begin{array}{cc} {\mathrm{TiO}}_{2}+h\nu & \to e^{-}+h^{+} \end{array}$$

(8) $$\begin{array}{cc} h^{+}+H_{2}O\left(\mathrm{ads}\right) & \to \;\cdot \;\mathrm{OH}+H^{+} \end{array}$$

(9) $$\begin{array}{cc} e^{-}+\mathrm{CNT} & \to \mathrm{enhanced}\;\mathrm{interfacial}\;\mathrm{transport} \end{array}$$

Equation (9) is schematic rather than stoichiometric, but it captures the widely invoked role of CNTs as interfacial highways for photogenerated carriers.

### 10.2. Architecture-Enabled Room-Temperature Sensing

A major objective in TiO_2_–CNT sensor development is the avoidance of high-temperature operation. TiO_2_–CNT hybrids are especially attractive in this regard because CNTs preserve conductivity under conditions where TiO_2_ alone often remains too resistive. Abdulhameed and Mahnashi achieved this through staged dielectrophoretic assembly; Seekaew, Hwang, Kumar, and others demonstrated different forms of room-temperature sensing for NH_3_, breath-acetone-related operation, and LPG/humidity-responsive behavior [7,14,22,26,49]. The shared lesson is that room-temperature performance depends less on the mere presence of CNTs than on whether the CNT network remains continuous and electronically meaningful after oxide integration.

### 10.3. Contacts, Arrays, and Signal Processing

Another important operational strategy is the deliberate use of contacts, temperature modulation, and array-level design to generate response diversity. Panzardi et al. showed that temperature-pulsed operation can improve sensing behavior in TiO_2_-decorated SWCNT networks [17]. Shooshtari and Salehi demonstrated that changing the electrode material can change the response pattern obtained from the same hybrid layer, thereby enriching virtual-array behavior [8]. Iwata et al. extended this concept further by using neural-network-based regression on TiO_2_ nanotube sensor arrays to predict gas concentrations from multisensor outputs [20]. These studies imply that TiO_2_–CNT platforms are especially promising when treated not as isolated films but as tunable nodes in multiparametric analytical systems. The fluctuation-enhanced sensing approach used by Drozdowska and Smulko adds another important dimension to this argument: in their Au–graphene–TiO_2_ device, resistance-noise spectra contained Lorentzian features whose intensity or corner frequency changed with NO_2_ concentration and with illumination wavelength, allowing the authors to distinguish Au-related plasmonic activation from TiO_2_-related UV activation [48]. Translating this strategy to TiO_2_–CNT sensors could help resolve a persistent weakness of the field, namely the tendency to infer the active interface from DC response alone rather than from independent dynamic or spectral signatures.

### 10.4. Flexibility and Self-Supporting Configurations

Mechanical flexibility is increasingly important for wearable, portable, and low-power sensing. Flexible and self-supporting TiO_2_–CNT architectures should not be regarded merely as packaging innovations. In many cases, they also reflect a more favorable distribution of oxide and CNT domains, improving gas accessibility and reducing mechanical failure. Chang’s interlaced film and the broader flexible TiO_2_-sensor literature surveyed by Nunes show that mechanically compliant device design can be compatible with high gas sensitivity when the hybrid is engineered from the outset rather than adapted from brittle oxide layers [16,27].

Ponnamma et al. provide a flexible platform directly relevant to sensing by embedding TiO_2_–CNT hybrid nanotubes into PVDF nanocomposites and evaluating both LPG sensing and piezoelectric energy harvesting [47]. The optimized PVDF/TiO_2_–CNT composite containing 2.5 wt% hybrid filler responded to 400 ppm LPG with a response time of 0.45 s, about nine times faster than analogous composites containing only TiO_2_ or only CNTs at the same filler loading [47]. Mechanistically, this result supports two points that are central to flexible TiO_2_–CNT sensors: the hybrid filler must be close to the percolation threshold to form an efficient conductive network, and the TiO_2_–CNT contact improves dispersion and interfacial charge transport more effectively than either filler alone. Because the PVDF matrix also contributes piezoelectric output, the study is particularly useful for the emerging concept of self-powered gas-sensing layers in which mechanical energy harvesting and chemiresistive gas response are coupled within one flexible film.

## 11. Practical Challenges Toward Real-World TiO_2_–CNT Gas Sensors

### 11.1. Commercial Baseline and Application-Relevant Performance Targets

A practical assessment of TiO_2_–CNT sensors requires comparison with technologies that are already deployed rather than with laboratory oxide devices alone. Commercial semiconducting metal-oxide (MOS) sensors remain attractive because they are inexpensive, compact, robust, and compatible with simple chemiresistive readout, although heater demand, cross-sensitivity, humidity dependence, and long-term calibration remain important limitations [93]. Representative commercial devices also show that “low power” is a moving target. A conventional Figaro TGS2600 uses a 210 mW heater while targeting low-ppm air contaminants, whereas the MEMS TGS8100 reduces heater power to about 15 mW for a similar nominal H_2_ detection range [94,95]. Likewise, the Bosch BME688 uses a micro-hotplate with a 320 °C heater target during continuous gas operation, but duty cycling can reduce the average current substantially at the cost of a longer response time [96]. Thus, room-temperature operation alone does not demonstrate an industrial advantage unless total system power, response time, calibration strategy, and environmental robustness are reported together.

Catalytic-bead (pellistor) sensors provide a different commercial baseline for combustible gases. The SGX VQ548MP operates its catalytic element at approximately 400–500 °C, draws about 34–42 mA at 3.0 V (roughly 0.10–0.13 W), and specifies a T_90_ below 12 s at 1% methane [97]. Its strengths are rapid, established detection in the percent-level combustible-gas regime and compatibility with certified safety instrumentation, whereas catalytic poisoning by silicones and sulfur-containing compounds remains a recognized failure mode [97]. TiO_2_–CNT hybrids should therefore not be presented as universal replacements for pellistors; their more credible opportunity lies in lower-temperature, lower-power, trace-gas, flexible, or optically assisted sensing, provided that these advantages survive realistic humidity, interferents, drift, and repeated-use testing.

Table 12 translates these commercial examples into an application-oriented baseline. Because MOS devices, pellistors, and TiO_2_–CNT chemiresistors use different response definitions, a universal numerical threshold for “response magnitude” would be misleading. Industrial relevance should instead be judged against the intended use case. A TiO_2_–CNT platform should demonstrate at least one clear system-level advantage—for example lower average power, lower thermal budget, lower detectable concentration, improved selectivity, or mechanical flexibility—without sacrificing response/recovery kinetics, calibration stability, humidity tolerance, batch reproducibility, or long-term durability. For photoactivated devices, the optical-source power must be included in the energy budget rather than reporting sensor-heater power alone.

### 11.2. Environmental Reliability, Durability, and Accelerated Stress Testing

Application readiness requires separating three phenomena that are often conflated in laboratory reports: reversible environmental interference, recoverable inhibition, and irreversible aging. Humidity is the best-documented TiO_2_–CNT example of the first category. Water can compete for adsorption sites, alter TiO_2_ hydroxylation, introduce protonic conduction, and perturb functionalized CNT junctions; consequently, a response measured only in dry gas does not establish useful selectivity in ambient service [7,26,37]. Hwang et al. showed this problem at the system level by requiring upstream dehumidification for breath-oriented acetone sensing [7]. Feasible mitigation strategies therefore include humidity conditioning or selective inlet layers, a colocated humidity reference, humidity-dependent calibration, and temperature- or light-modulated operation when these approaches are compatible with the intended application.

Poisoning must be treated more cautiously because direct TiO_2_–CNT durability data are sparse. Sulfur-containing species are relevant both as target analytes and as potential strongly adsorbing interferents, whereas organosiloxanes are established poisons for catalytic combustible-gas sensors [97]. These observations should not be transferred uncritically to TiO_2_–CNT hybrids. Instead, they define a validation gap: sensors intended for industrial or indoor deployment should be challenged with application-relevant sulfur compounds, silicone vapors where present, and moisture, followed by clean-air recovery, recalibration, and post-exposure structural/electrical characterization. A loss that disappears after purge or controlled regeneration should be distinguished from permanent sensitivity loss or baseline displacement.

Long-term drift can originate from multiple levels of the device. In conventional conductometric metal-oxide sensors, documented causes include structural or phase transformation, poisoning, contact and heater degradation, temperature/humidity fluctuations, and other environmental disturbances [98]. Long-term field studies of commercial MOS arrays likewise show that nominally identical sensors can drift differently over years of operation [99]. Conversely, a sprayed SWCNT NH_3_ sensor retained up to 96% of its response after four years of ambient storage, demonstrating that CNT networks can be intrinsically stable under some conditions [100]. This CNT result is encouraging but does not establish equivalent stability for TiO_2_–CNT interfaces, where oxide coarsening, changes in functional groups, contact resistance, catalyst residues, and interface reconstruction may introduce additional failure pathways. Hybrid-specific aging therefore remains an evidence gap rather than a demonstrated advantage.

Accelerated environmental testing should be matched to the intended package and deployment. A severe 85 °C/85% RH exposure, for example, is widely used as a temperature–humidity qualification-style stress for non-hermetic electronic devices [101]; for TiO_2_–CNT sensors it should be regarded as an accelerated durability screen rather than a universal operating condition. Where such a stress exceeds the limits of a flexible substrate, adhesive, or package, application-specific temperature/RH cycling is more informative. In either case, response magnitude alone is insufficient: baseline resistance, calibration slope, response/recovery time, hysteresis, noise, and structural integrity should be compared before and after stress.

Flexible devices require an additional mechanical reliability layer. A robust report should specify the bend radius, strain direction, number of cycles, and whether gas response was measured during or after mechanical fatigue. Baseline resistance and sensitivity should be tracked together because a mechanically induced change in CNT percolation can mimic a chemical response. Post-fatigue microscopy or resistance mapping can then distinguish reversible strain modulation from cracking, delamination, electrode damage, or permanent loss of CNT–CNT and TiO_2_–CNT contacts. Table 13 summarizes a minimum validation set for these real-device failure modes.

The recurring limitations identified in the literature are summarized in Table 14. Figure 3 provides a complementary hierarchy for judging whether a reported TiO_2_–CNT sensor has moved from a proof-of-concept response toward a reproducible, mechanistically supported sensing platform.

Despite rapid progress, the TiO_2_–CNT sensing literature still contains several methodological weaknesses that hinder the emergence of broadly applicable design rules. These weaknesses are not minor reporting issues. They directly affect whether a proposed mechanism is credible and whether a reported sensor can be compared with the rest of the field.

The first limitation is metric comparability. Response may be defined as $R_{a}/R_{g}$, $R_{g}/R_{a}$, ΔR/R, wavelength shift, resonance shift, frequency shift, or prediction error. In hybrid TiO_2_–CNT layers, the response sign may also change depending on whether oxide domains, CNT junctions, or interfacial barriers dominate the current pathway. Without explicit normalization and baseline conventions, even sensors targeting the same analyte become difficult to compare [3,5].

The second limitation is environmental incompleteness. Humidity is often reported as a secondary condition or omitted entirely, even though water competes for TiO_2_ adsorption sites, modifies hydroxylation, changes proton availability, and perturbs functionalized CNT junctions. For NH_3_, acetone, breath-related VOCs, and room-temperature NO_2_, uncontrolled humidity can be large enough to mask the intrinsic analyte response. Therefore, humidity should be treated as a first-order experimental variable rather than as background metadata.

The third limitation is weak mechanistic validation. Many papers invoke charge transfer, oxygen vacancies, heterojunction formation, catalytic spillover, increased active area, or improved electron extraction from performance trends alone. Such explanations are plausible but not necessarily proven. A stronger mechanistic claim requires at least one independent line of evidence, such as XPS shifts, Raman changes, impedance spectra, photoconductivity, Kelvin-probe measurements, noise spectroscopy, in situ spectroscopy, or systematic control devices [9,31,35].

The fourth limitation is insufficient reproducibility assessment. Many studies provide attractive first-cycle responses but little information on drift, storage stability, batch-to-batch variation, repeated bending for flexible devices, or fabrication tolerance. This is particularly serious for CNT-based systems, where purification history, residual catalyst, acid treatment, dispersion protocol, surfactant residues, and drying route can all alter the final percolation network. Unless such variables are controlled and reported consistently, interlaboratory comparability will remain limited.

Finally, much of the literature still emphasizes proof-of-concept response over application-ready benchmarking. Interferent panels are often narrow, long-term cycling is limited, and practical issues such as power budget, recovery completeness, calibration stability, and tolerance to fluctuating real atmospheres remain underexplored. A serious comprehensive review must therefore distinguish clearly between promising materials and deployable sensing strategies.

A final gap is the distance between material-level performance and deployable sensor systems. Most TiO_2_–CNT reports remain at the proof-of-concept stage, where a single sensing film is exposed to a controlled analyte under laboratory flow. Far fewer studies integrate humidity conditioning, calibration transfer, temperature compensation, sensor-to-sensor statistics, packaging, wireless readout, or field-relevant mixtures. The electronic-nose study of Shooshtari et al. and the breath-acetone system of Hwang et al. are notable because they move beyond the isolated sensing film by incorporating, respectively, multivariate signal processing and upstream humidity management [7,8]. Future progress should therefore be judged not only by lower LOD or higher response, but also by whether the sensing layer survives the system-level constraints required for a repeatable measurement outside the laboratory.

## 12. Design Rules and Outlook

As synthesized in Figure 3 and Figure 4, the most useful future reports will combine response magnitude with controls, environmental realism, mechanistic validation, and application-oriented design. The literature surveyed here indicates that future progress in TiO_2_–CNT gas sensors will depend less on discovering entirely new ingredients and more on controlling how known ingredients are integrated, tested, and interpreted. Several design rules emerge consistently from the core corpus.

These rules are intended to be applied after the four benchmarking gates introduced in Section 5: first establish comparability of test conditions, then verify the evidence for the architecture claim, then assess application constraints, and finally test the proposed mechanism independently. A design rule should therefore be considered actionable only when the corresponding sensor passes the relevant gate or when the missing validation is stated explicitly as a required next experiment.

The first rule is deliberately negative: there is no universally optimal TiO_2_–CNT morphology. Marichy et al. found a thin continuous ALD coating superior to discontinuous coverage for NO_2_/O_2_, whereas Kaushik et al. showed that increasing TiO_2_ thickness from a thin ALD layer penalized NH_3_ sensing [29,30]. Santangelo et al. likewise demonstrated a non-monotonic optimum in CNT loading for H_2_ sensing [28]. These studies are more informative than isolated record responses because they deliberately vary one structural parameter and thereby provide stronger evidence of causality. Accordingly, future work should prioritize controlled series—oxide thickness, CNT fraction, functionalization density, illumination intensity or humidity—over single-point optimization.

**Rule** **1.** **Optimize interfacial electrical leverage, not nominal composition.** The strongest evidence does not support a universal preference for nanoparticle decoration, partial coverage, or conformal shells. Instead, the optimum occurs when gas-induced changes in TiO_2_ surface charge or barrier height remain strongly coupled to an electrically sensitive CNT network. Thin continuous ALD coatings can outperform discontinuous coverage in one analyte/temperature window, whereas thicker coatings can penalize room-temperature NH_3_ response in another [29,30]. Architecture should therefore be optimized through controlled thickness and loading series rather than selected by morphology label alone.
**Rule** **2.** **Choose the synthesis route according to the desired architecture.** Sol–gel and hydrothermal methods remain valuable for scalable porous hybrids, but ALD, staged DEP assembly, and controlled shell growth are more suitable when the goal is to test interfacial thickness, shell sequence, or contact quality [10,11,12,15,32,102]. Carbon pretreatment should be reported explicitly because oxygenated groups such as ϕ-COOH can strongly modify TiO_2_ nucleation, charge redistribution, and interfacial stability.
**Rule** **3.** **Let the analyte determine the architecture.** Oxidizing gases such as NO_2_ benefit strongly from accessible oxide domains connected to low-resistance CNT pathways. Reducing gases such as H_2_, NH_3_, and VOCs often require a more delicate balance between adsorption strength, recovery, humidity tolerance, and catalytic or photoactivated assistance. There is therefore unlikely to be a single universally optimal TiO_2_–CNT geometry.
**Rule** **4.** **Treat humidity and illumination as design variables.** Water content, wavelength, light intensity, thermal budget, and recovery protocol can change the dominant sensing mechanism. They should be controlled, reported, and discussed as part of the sensor architecture rather than as auxiliary operating details [8,20,24,25].
**Rule** **5.** **Do not claim a mechanism from response trends alone.** Charge transfer, oxygen vacancies, heterojunctions, spillover, and photocarrier extraction are credible only when supported by independent evidence. Future studies should combine chemiresistive measurements with spectroscopy, impedance, photoconductivity, Kelvin-probe, noise, or operando experiments whenever possible.
**Rule** **6.** **Benchmark against practical failure modes.** A serious next-generation TiO_2_–CNT sensor study should include humidity-controlled tests, repeated cycling, interferent analysis, explicit response definitions, statistically meaningful reproducibility data, and post-test stability assessment. Without these elements, even highly promising sensors will remain difficult to compare and difficult to translate.

Taken together, these design guidelines indicate that progress in TiO_2_–CNT gas sensors should be evaluated through the quality of interface control, the completeness of benchmarking protocols, and the strength of mechanistic validation. Rather than treating high response values as isolated performance indicators, future studies should connect synthesis route, hybrid architecture, analyte chemistry, activation strategy, humidity response, and device stability within a single structure–performance framework. This perspective provides the basis for the final conclusions and future directions summarized below.

## 13. Conclusions

This comprehensive review shows that TiO_2_–CNT gas sensors are best understood as interfacial transport systems rather than as simple mixtures of an oxide receptor and a conductive carbon additive. Gas adsorption changes surface charge, oxygen coverage, defect occupation, and interfacial barriers, but the measured signal is determined by how those local perturbations propagate through TiO_2_ domains, TiO_2_–CNT contacts, CNT–CNT junctions, and metal contacts. The response polarity, magnitude, and recovery kinetics therefore cannot be assigned reliably from the nominal semiconductor type of TiO_2_ or CNTs alone.

Three conclusions are especially robust. First, architecture dominates nominal composition. Thin conformal coatings, nanoparticle decoration, interpenetrating networks, and interlaced films can each be effective when they preserve both accessible receptor sites and an electrically sensitive current path. Direct thickness- and loading-dependent studies show that neither maximum oxide coverage nor maximum CNT content is a universal optimum. Second, low-temperature operation is frequently enabled by CNT transport, catalytic modification, or photoactivation, but these gains must be evaluated together with recovery, humidity dependence, and the energy required for optical or thermal regeneration. Third, cross-study performance rankings remain fragile because response equations, concentration ranges, humidity, flow conditions, and activation protocols are rarely standardized.

The field is therefore mature enough to move beyond demonstrations of “enhanced response.” The next generation of TiO_2_–CNT studies should report complete material descriptors, explicit response definitions, calibration slopes with uncertainty, response/recovery criteria, humidity and interferent testing, repeated cycles, long-term drift, and sensor-to-sensor variability. Mechanistic assignments should be supported by controlled architecture series and, where possible, operando or in situ evidence rather than inferred solely from ex situ band diagrams.

The most promising path toward practical devices is not a single composition but a design workflow: define the analyte and deployment environment; engineer the TiO_2_ receptor chemistry; control the TiO_2_–CNT interface and percolation network; select the minimum activation energy compatible with recovery; and validate the complete device under realistic humidity, interference, and stability constraints. Applying this framework should allow TiO_2_–CNT hybrids to progress from heterogeneous proof-of-concept reports toward reproducible, low-power, and application-relevant gas-sensing platforms.

## Figures and Tables

**Figure 1:**
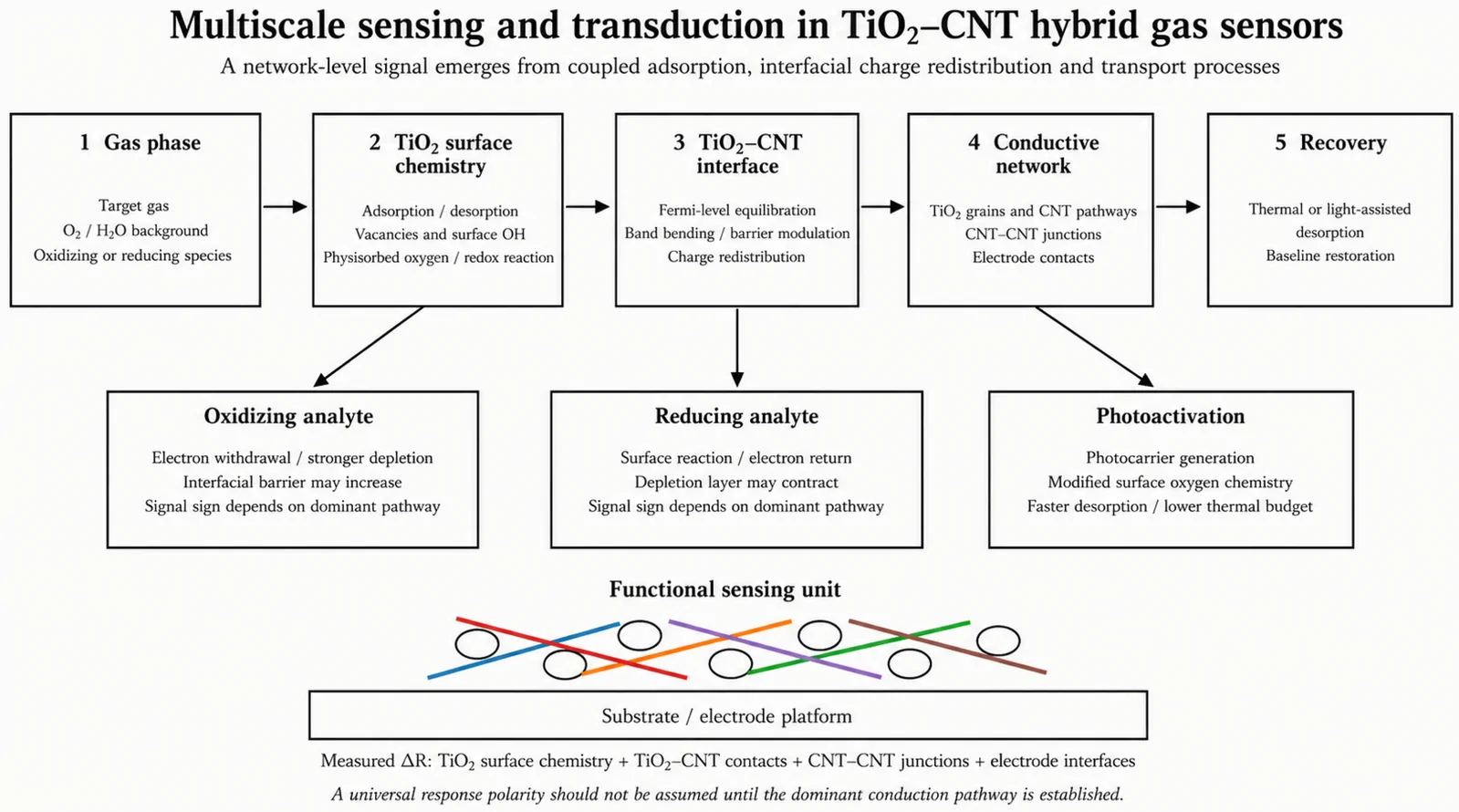
Multiscale sensing and transduction pathways in TiO_2_–CNT hybrid gas sensors. Colored lines schematically represent CNT conductive pathways within the hybrid sensing network. Response polarity depends on the dominant conduction pathway.

**Figure 2:**
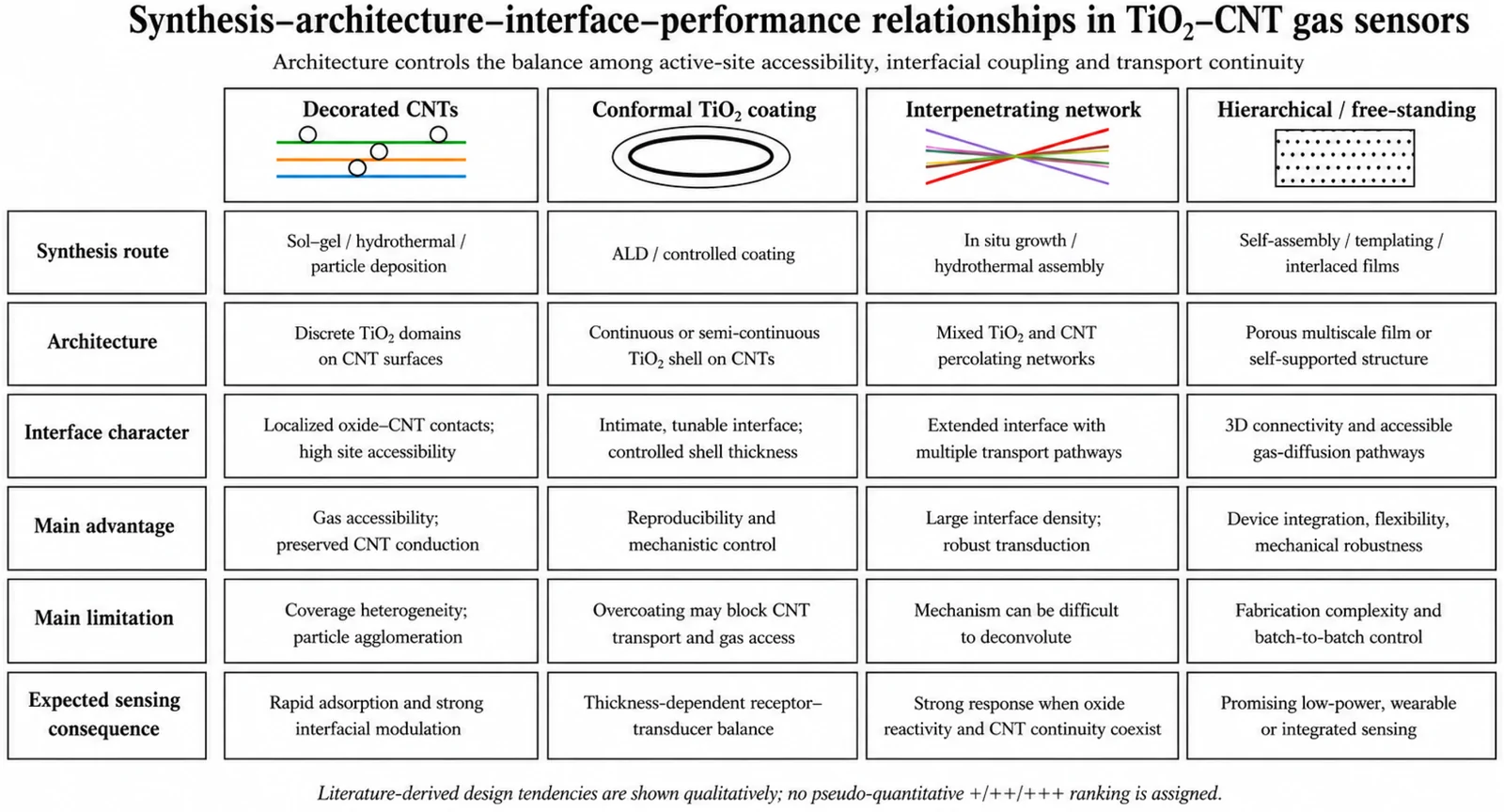
Synthesis–architecture–interface–performance relationships in TiO_2_–CNT gas sensors. Colored lines schematically represent CNT pathways or networks within the different hybrid architectures and do not encode quantitative performance. Trends are qualitative rather than numerical rankings.

**Figure 3:**
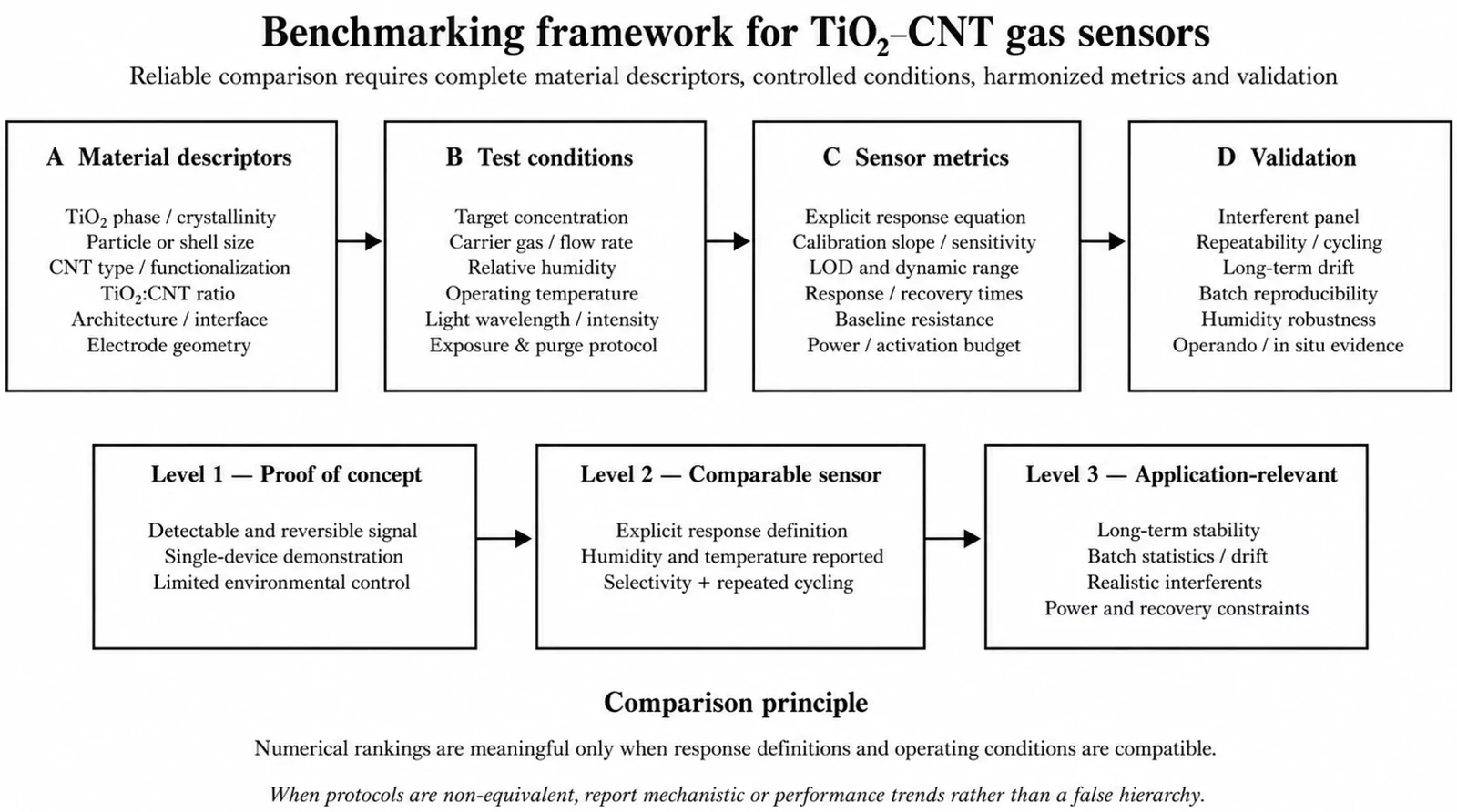
Benchmarking framework for TiO_2_–CNT gas sensors. Comparisons require compatible response definitions and operating conditions.

**Figure 4:**
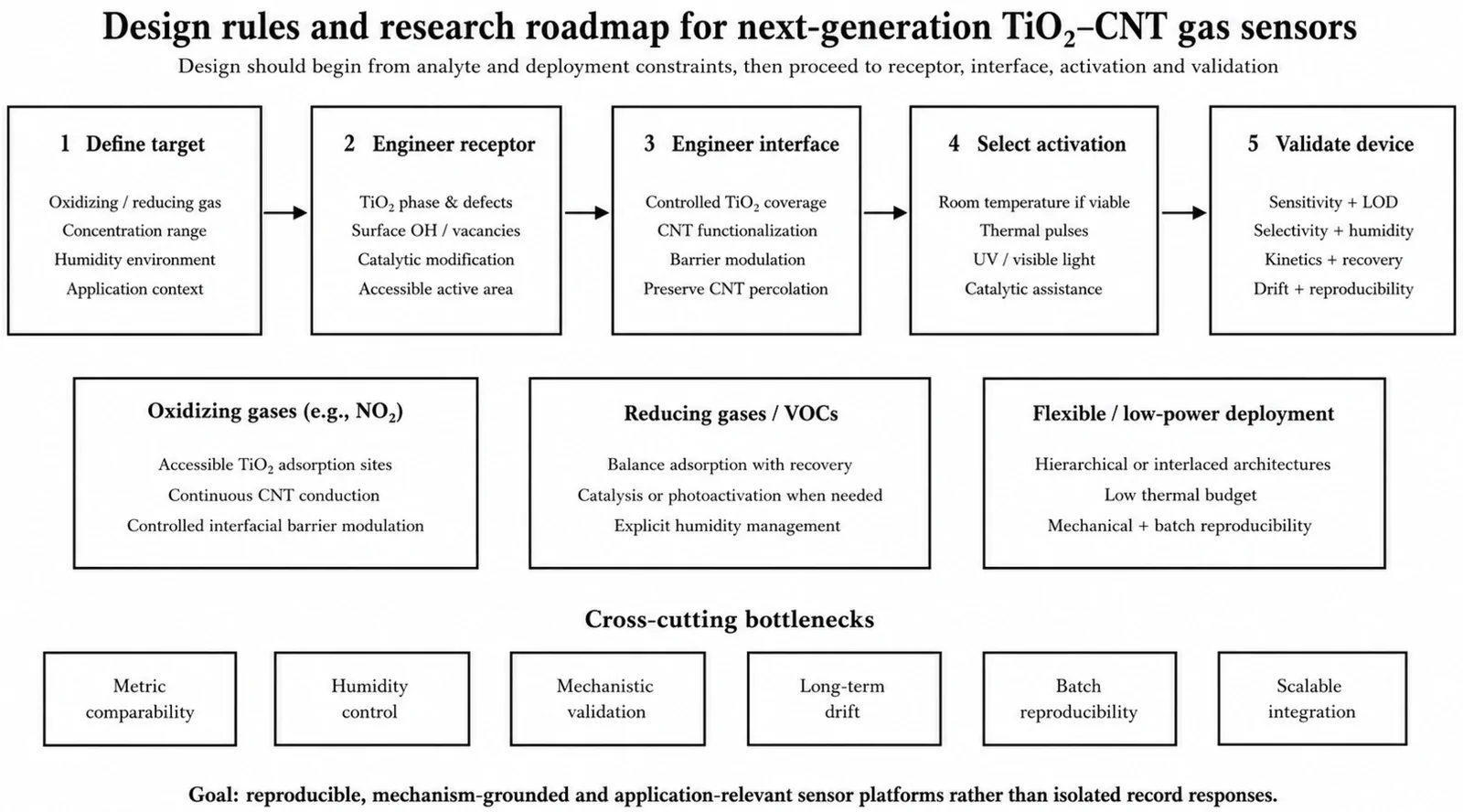
Design rules and research roadmap for TiO_2_–CNT gas sensors. The workflow links analyte, receptor, interface, activation, and device validation.

**Table 1:** Abbreviations and symbols used in TiO_2_–CNT gas-sensor literature.

Abbreviation or Symbol	Definition
Materials and architectures	
TiO_2_	Titanium dioxide
CNTs	Carbon nanotubes
SWCNTs	Single-walled carbon nanotubes
MWCNTs	Multi-walled carbon nanotubes
TiO_2_–CNT	Hybrid material or device containing titanium dioxide and carbon nanotubes
SWCNT@TiO_2_	Interfacial architecture in which TiO_2_ is anchored, deposited, or grown on the surface of single-walled carbon nanotubes; the “@” symbol denotes an interfacial configuration rather than a simple physical mixture
GO	Graphene oxide
rGO	Reduced graphene oxide
MOF	Metal–organic framework
Analytes and operating conditions	
NO_*x*_	Nitrogen oxides, principally NO and NO_2_
VOCs	Volatile organic compounds
NH_3_	Ammonia
H_2_	Hydrogen
H_2_S	Hydrogen sulfide
HCHO	Formaldehyde
O_2_	Molecular oxygen
LPG	Liquefied petroleum gas
RH	Relative humidity
RT	Room temperature
UV	Ultraviolet radiation
Synthesis and computational methods	
ALD	Atomic layer deposition
CVD	Chemical vapor deposition
PECVD	Plasma-enhanced chemical vapor deposition
DEP	Dielectrophoresis
DFT	Density functional theory
Characterization and mechanistic analysis	
XPS	X-ray photoelectron spectroscopy
XRD	X-ray diffraction
SEM	Scanning electron microscopy
TEM	Transmission electron microscopy
FTIR	Fourier-transform infrared spectroscopy
EPR	Electron paramagnetic resonance
BET	Brunauer–Emmett–Teller surface-area analysis
EDL	Electron depletion layer
Gas-sensing metrics and symbols	
LOD	Limit of detection
$R_{a}$	Electrical resistance measured in air or in the reference atmosphere
$R_{g}$	Electrical resistance measured in the target-gas atmosphere
$R_{a}/R_{g}$	Resistance-ratio response convention commonly used when the resistance decreases after exposure to the target gas
$R_{g}/R_{a}$	Resistance-ratio response convention commonly used when the resistance increases after exposure to the target gas
ΔR/R	Relative resistance change; the precise reference resistance should be defined for each study
$\tau_{\mathrm{res}}$	Response time
$\tau_{\mathrm{rec}}$	Recovery time

**Table 2:** Evidence hierarchy adopted in this review.

Evidence Level	Included Studies	Use in This Review
Core evidence	TiO_2_–CNT or TiO_2_–CNT-containing sensors with explicit response, target gas, operating condition, and transduction data	Used to derive analyte-specific structure–performance relationships and practical design rules
Supporting evidence	TiO_2_–CNT studies in photoelectrochemical, photocatalytic, optoelectronic, or interfacial-engineering contexts	Used only when they clarify oxide nucleation, CNT functionalization, charge extraction, shell continuity, recombination, or loading optima
Contextual analogues	TiO_2_–graphene/rGO, TiO_2_–metal, TiO_2_–oxide, or CNT sensors without TiO_2_	Used as mechanistic comparators, not as direct proof of TiO_2_–CNT gas-sensor performance
Excluded or down-weighted evidence	Reports lacking operating conditions, response definitions, controls, or interpretable material description	Not used to support quantitative benchmarking or causal mechanistic claims

**Table 3:** Key physicochemical variables in TiO_2_–CNT gas sensors.

Variable	Physicochemical Effect	Sensing Implication
TiO_2_ crystallinity and phase [1,4]	Changes defect chemistry, adsorption behavior, and oxide transport	Alters sensitivity, stability, and recovery
CNT type and purification history [3,6]	Modifies percolation, work function, and junction resistance	Shifts baseline resistance and charge-transfer efficiency
CNT functionalization degree [7,13,31]	Improves oxide anchoring but increases defects and water affinity	May enhance hybridization while raising humidity sensitivity
TiO_2_ coverage continuity [10,11,15]	Controls accessibility of active sites and preservation of CNT pathways	Defines the trade-off between selectivity and conductivity
Oxygen-vacancy density [9,34,35]	Changes adsorption energetics and carrier trapping	Modulates response magnitude and activation threshold
Humidity level [7,26,37]	Alters hydroxylation, competitive adsorption, and protonic contributions	Changes baseline, selectivity, and recovery kinetics
Photoactivation mode [19,24,25]	Generates additional carriers and modifies surface chemistry	Enables low-temperature sensing and faster recovery

**Table 4:** Structure–performance relationships in TiO_2_–CNT gas sensors.

Structural Variable	Potential Benefit	Main Risk	Design Implication
Partial TiO_2_ decoration [6,16,18]	Adds oxide receptor sites while preserving CNT transport	Insufficient oxide can give weak analyte interaction	Optimize coverage near the point where adsorption increases without interrupting percolation
Conformal TiO_2_ shell [11,15,39]	Provides a controlled interface and reproducible surface chemistry	Thick shells can encapsulate CNTs and create transport bottlenecks	Match shell thickness to depletion length, gas accessibility, and baseline resistance
CNT functionalization [7,13,32]	Improves dispersion, oxide nucleation, and interfacial bonding	Over-oxidation can damage conductivity and increase water sensitivity	Report pretreatment conditions and optimize functional-group density
Hierarchical porosity [11,43,44]	Enhances gas diffusion and recovery	Excessive porosity may weaken mechanical stability or reproducibility	Preserve open pathways from the gas phase to oxide and interfacial sites
Ternary sensitization [15,19,25]	Can improve selectivity, lower the activation requirement, or enhance light harvesting	The mechanism becomes difficult to deconvolute	Use the added phase only when it addresses a defined limitation and include appropriate controls
Photoactivation [16,19,24,25]	Accelerates recovery and enables lower-temperature operation	The response depends on wavelength, intensity, and possible photochemical side reactions	Treat illumination as an operational variable rather than as a generic auxiliary condition

**Table 5:** Cross-comparison of direct sensing studies that reveal non-universal architecture optima.

Study	Direct Observation	Apparent Contradiction	Interpretation in This Review
Marichy et al. [29]	NO_2_: 5 and 8 ppm in dry air; RT–250 °C (architecture/thickness comparison at 150 °C); O_2_ also evaluated; no optical activation. A continuous TiO_2_ coating of about 5.5 nm gave higher response than discontinuous coating or nanoparticle decoration.	Continuous coverage can outperform discrete decoration.	A thin continuous shell can maximize interfacial coupling without eliminating CNT-network transduction.
Kaushik et al. [30]	NH_3_ at room temperature (25 ± 2 °C); 100, 250, and 500 ppm; RH 1–5%; no optical activation. A thin ALD TiO_2_ coating gave the strongest response; CNT functionalization also modified nucleation and response.	Increasing oxide thickness does not monotonically improve sensing.	The optimum depends on a receptor–transducer balance and on how the oxide nucleates on the CNT scaffold.
Santangelo et al. [28]	H_2_: 5–100% in He; near-room- temperature operation; inert He carrier; no optical activation. Response varied non-monotonically with CNT fraction, with an optimum near 34 wt%.	Maximum CNT loading is not equivalent to maximum electrical benefit.	Percolation, oxide stoichiometry, agglomeration, and TiO_2_–CNT contact must be optimized together.
Ito et al. [18]	NO_2_: tested at 5 ppm in synthetic air; room temperature; RH not reported; no optical activation. Response depended on TiO_2_ nanoparticle size and loading.	Maximum oxide loading is not necessarily the optimum.	Excess oxide can reduce the electrical leverage of CNT transport or alter gas-accessible interfacial area.

**Table 6:** Recommended benchmarking fields for TiO_2_–CNT gas sensors.

Category	Minimum Information	Why It Matters
Material identity [1,3,5]	TiO_2_ phase, crystallinity, CNT type, functionalization, oxide loading, shell thickness, or particle size	Separates nominal composition from the actual interfacial architecture
Architecture [2,16,27]	Decoration, partial shell, conformal shell, interlaced film, nanotube array, membrane, or electrode-integrated layer	Determines whether current flows mainly through oxide domains, CNT pathways, or TiO_2_–CNT contacts
Reference devices [3,5,47]	Pristine TiO_2_, pristine CNTs, physical mixture, and true hybrid prepared using comparable processing conditions	Prevents over-attribution of performance improvements to heterojunction or interfacial effects
Operating conditions [16,24,26,27]	Temperature, relative humidity, gas flow, analyte concentration range, carrier gas, and illumination wavelength and intensity	Controls adsorption, oxygen chemistry, photoactivation, response, and recovery
Response definition [3,5]	$R_{a}/R_{g}$, $R_{g}/R_{a}$, ΔR/R, direction of resistance change, baseline convention, and recovery criterion	Allows meaningful comparison among n-type oxide, p-type CNT, and mixed-conduction sensors
Kinetics and reversibility [16,17,27]	Response time, recovery time, cycle-to-cycle drift, incomplete recovery, and baseline stabilization time	Distinguishes a large transient response from reproducible and deployable sensing behavior
Selectivity and robustness [5,26,27]	Interferent tests, humidity cycling, long-term stability, batch reproducibility, and repeated bending for flexible devices	Determines whether the material is a practical sensor candidate or only a proof-of-concept sensing layer
Mechanistic evidence [9,10,31,48]	XPS, Raman spectroscopy, impedance, Kelvin-probe measurements, photoconductivity, noise spectra, and in situ or operando spectroscopy	Tests whether the proposed charge-transfer, vacancy, or heterojunction mechanism is causal rather than inferred only from response trends

**Table 7:** Minimum controls for mechanistic claims in TiO_2_–CNT gas sensors.

Claim	Minimum Controls	Diagnostic Evidence	Risk If Omitted
Analyte-dependent interfacial charge transfer [9,31,48]	TiO_2_-only, CNT-only, physical mixture, and true hybrid prepared under comparable conditions	XPS binding-energy shifts, Kelvin-probe/work-function data, impedance spectra, photoconductivity, or noise analysis	Improved response may be incorrectly assigned to the interface rather than to surface area or film resistance
Oxygen-vacancy-driven sensing [34,35,49]	Vacancy-poor and vacancy-rich TiO_2_–CNT samples with similar morphology and loading	XPS O 1s/Ti 2p analysis, Raman disorder, EPR, photoluminescence quenching, or controlled annealing studies	Defect chemistry may be inferred only from response magnitude
CNT functionalization effect [7,13,32,36]	Pristine, mildly oxidized, and strongly oxidized CNT scaffolds with the same oxide deposition route	Raman D/G ratio, XPS functional groups, zeta potential or dispersion stability, sheet resistance, and humidity response	Better oxide anchoring may be confused with damaged percolation or stronger water adsorption
Photoactivation mechanism [16,19,25,48]	Dark, UV/visible, intensity-dependent, and wavelength-dependent measurements	Photocurrent transients, recovery kinetics, wavelength dependence, and temperature-controlled light-on/light-off tests	Faster recovery may be mistaken for a specific TiO_2_–CNT photocarrier pathway
Catalytic or ternary sensitization [6,15,19,25]	Binary TiO_2_–CNT, additive-only, and ternary hybrid devices	Selectivity panel, activation-energy trend, surface-state analysis, and post-test structural verification	Added complexity may produce response enhancement without a clear causal mechanism
Humidity tolerance [7,26,37]	Dry, controlled-RH, humidity-only, and analyte-plus-humidity protocols	Baseline drift, RH cycling, impedance frequency response, and recovery completeness	Apparent selectivity may actually reflect water-modulated transport

**Table 8:** Recommended operando and in situ measurements for mechanistic verification of proposed sensing mechanisms in TiO_2_–CNT systems.

Claim to Test	Most Informative Experiment	What Would Constitute Stronger Evidence
Interfacial charge transfer/barrier modulation	Gas-synchronized Kelvin-probe or KPFM/work- function measurements, impedance spectroscopy, and electrical response; operando or near- ambient-pressure XPS where feasible [50,51,52].	A reversible work-function, interfacial-potential, or impedance change that follows analyte exposure and tracks the electrical signal rather than only a before/after shift.
Oxygen-vacancy involvement	EPR and in situ/near-ambient-pressure XPS under controlled oxygen/analyte atmospheres, combined with deliberately vacancy-poor and vacancy-rich samples [53,54].	A controlled change in vacancy-related states that covaries with sensing response while morphology and loading remain comparable; an O 1s fit alone is insufficient.
Catalytic spillover or surface-intermediate transfer	Operando DRIFTS/FTIR or Raman spectroscopy during gas exposure, ideally with isotopic labeling and outlet mass spectrometry, synchronized with resistance [55,56].	Identification and time-resolved evolution of surface intermediates on the relevant phase, with kinetics consistent with the onset and recovery of the sensor signal.
Photoactivated transduction	Transient photoconductivity/photocurrent together with wavelength- and intensity- dependent sensing, light-on/light-off kinetics, and operando optical or vibrational spectroscopy [57].	A reproducible spectral and intensity dependence linking photocarrier generation or surface photochemistry to the sensing and recovery kinetics.
Adsorption/desorption-limited recovery	Time-resolved DRIFTS/FTIR, temperature- programmed desorption or outlet mass spectrometry combined with pulsed gas exposure and electrical readout [50,54].	Desorption or surface-species decay that quantitatively follows electrical recovery and distinguishes slow surface chemistry from slow charge transport.

**Table 9:** Worked application of the benchmarking framework to the CNT/anatase-TiO_2_ NO sensor reported by Chang et al. [16].

Benchmarking Field	Evidence Demonstrated in the Study	Interpretation Under the Present Framework
Material/architecture	Interlaced CNT/anatase-TiO_2_ self-supporting flexible film.	Architecture is explicit and directly linked to combined sensing and flexibility.
Target/response	NO: 0.5–200 ppm; room temperature; RH not reported; UV-assisted recovery; reported LOD 500 ppb. A 41% resistance change was reported at 50 ppm.	Strong proof-of-concept response, but the value should be compared only with studies using compatible concentration and response definitions.
Selectivity/recovery	Selectivity tested against NH_3_, NO_2_, CH_4_, and H_2_S; reversible recovery assisted by UV irradiation.	Supports analyte discrimination and reversibility; UV conditions must enter the power/activation benchmark.
Mechanical validation	Sensing behavior retained under complex deformation of the interlaced film.	Promising flexible-device evidence; defined bend radius, cycle count, and post-fatigue statistics would improve comparability.
Application-level fields still needed	Humidity dependence, total optical energy budget, long-term drift, batch statistics, and standardized fatigue testing.	These fields separate a strong laboratory demonstration from an application-relevant Level-3 validation.

**Table 10:** Representative TiO_2_–CNT systems for NO_*x*_ sensing.

Reference	Architecture	Target/Operation	Main Advantage	Main Limitation
Marichy et al. [29]	ALD TiO_2_-coated CNTs	NO_2_: 5 and 8 ppm in dry air; RT–250 °C (architecture comparison at 150 °C); O_2_: 2.5, 5, 10, and 20% in N_2_ also evaluated; no optical activation	A continuous TiO_2_ coating of approximately 5.5 nm gave higher response than discontinuous films or nanoparticle decoration and exposed p-type network behavior.	The result is strongly thickness- and architecture-dependent, and the p-type response cannot be interpreted from TiO_2_ semiconductor type alone.
Chang et al. [16]	Interlaced CNT/TiO_2_ film	NO: 0.5–200 ppm; room temperature; RH not reported; UV-assisted recovery; reported LOD 500 ppb	Flexible conductive network with accessible oxide domains; useful example of mechanically robust room-temperature NO sensing.	Recovery still depends on optical assistance, so the system is not fully passive under dark room-temperature conditions.
Fort et al. [6]	TiO_2_-decorated SWCNT network (Au-decorated SWCNTs as comparator)	NO_2_, 12.5–50 ppm; 120–240 °C (TiO_2_-decorated optimum about 220 °C); dry and humid air (RH = 25% at 25 °C); cross-sensitivity evaluated; no optical activation.	TiO_2_-decorated SWCNTs showed enhanced NO_2_ sensitivity, particularly at lower temperature; the optimum temperature was about 220 °C, although TiO_2_ decoration increased humidity cross-sensitivity.	TiO_2_ decoration also increased RH cross-sensitivity, and the Au- and TiO_2_-decorated devices should be interpreted as separate architectures rather than as a single ternary mechanism.
Panzardi et al. [17]	TiO_2_-decorated SWCNT network	NO_2_: 12, 25, and 37 ppm in dry synthetic air; optimized pulsed profile 220/80 °C; no optical activation	Temperature-pulsed operation improves dynamic response and provides mechanistic insight into adsorption and desorption kinetics.	Quantitative comparison with isothermal sensors depends strongly on the selected pulse profile and operating protocol.
Ito et al. [18]	CNT/TiO_2_ p–n heterojunction	NO_2_: tested at 5 ppm in synthetic air; room temperature; RH not reported; no optical activation	Clear demonstration that TiO_2_ amount and nanoparticle size affect the NO_2_ response of CNT/TiO_2_ heterojunction sensors.	Excess oxide can lower sensitivity by disrupting CNT transport or reducing the effectiveness of interfacial modulation.
Wang et al. [63]	TiO_2_ NTs/CNTs composite	NO_2_: 2, 4, 6, 8, and 10 ppm; 120 °C; UV-assisted sensing; RH not systematically evaluated	UV-assisted operation improves the response toward NO_2_ and connects the sensing material with a practical monitoring scenario.	The application-specific platform is useful but less general as a benchmark for broad NO_*x*_ sensor comparison.
Drozdowska and Smulko [48]	Ink-printed Au–graphene–TiO_2_ hybrid	NO_2_: 1–7 ppm at RT (∼25 °C); RH 40% tested at 5 ppm; 275 nm UV and 515 nm green illumination (∼2 mW cm^−2^); 10 ppb DC detection limit under UV	Useful analogue for wavelength-selective activation: UV addresses TiO_2_-photoactive regions, green light addresses Au-plasmonic regions, and noise spectra help identify the active interfacial domain.	Graphene replaces CNTs, so the study is best used as a mechanistic analogue for TiO_2_–carbon sensing rather than as a direct TiO_2_–CNT benchmark.
Ling et al. [61]	TiO_2_ nanotube arrays/rGO heterojunction nanocomposites	NO_2_: 1–50 ppm; room temperature; RH not reported; no optical activation; LOD 1 ppm	Shows that a small rGO fraction can bridge TiO_2_ nanotubes, increase chemisorbed oxygen, and produce a strong response through TiO_2_/carbon heterojunctions.	The carbon phase is rGO rather than CNTs, and the best response requires an optimized carbon loading rather than maximum conductive-additive content.
Choi et al. [60]	ZnO hemitubes or nanotubes decorated with TiO_2_ nanoparticles	NO_2_: 5, 10, 25, and 50 ppm; 20 ± 10 °C; RH 50 ± 20%; UV photoactivation (approximately 2.0 W)	Clear demonstration that TiO_2_ nanoparticle decoration and UV photoactivation can enable room-temperature response through barrier and depletion-layer modulation.	The platform is ZnO/TiO_2_ rather than TiO_2_–CNT, so it should be used as a mechanistic analogue rather than a direct carbon-hybrid benchmark.

**Table 11:** Cross-analyte design matrix for TiO_2_–CNT gas sensors.

Analyte Class	Design Emphasis	Main Risk	Critical Benchmark
NO_*x*_ [16,18]	Partial TiO_2_ decoration, interlaced films, accessible oxide domains, and preservation of continuous CNT transport pathways	Excessive oxide loading or continuous coverage can increase baseline resistance, restrict gas access, and slow recovery	Response and recovery at low concentration under controlled and application-relevant humidity
NH_3_ [15,22]	Defect-rich or functionalized oxide surfaces coupled to stable CNT percolation; the chemistry and conductivity of the outermost oxide layer should be controlled	Competitive adsorption of NH_3_ and water can produce baseline drift, cross-sensitivity, and apparent changes in selectivity	Humidity-dependent selectivity, repeated response–recovery cycles, and comparison of different outer-shell compositions
H_2_ [14,68]	Staged assembly, catalytic or semiconducting activation, and preservation of a low-resistance CNT conduction pathway	Random mixing, agglomeration, or inappropriate binder processing can interrupt CNT percolation and obscure the contribution of the interface	Comparison with TiO_2_-only, CNT-only, physical-mixture, and processing-matched hybrid devices
H_2_S [21]	Chemically robust TiO_2_–CNT interfaces and, where appropriate, optical, plasmonic, or multiparametric transduction	Strong sulfur adsorption or surface reaction may impair reversibility, cause poisoning, or reduce discrimination against other reducing gases	Recovery completeness, poisoning resistance, repeated cycling, and a broad interferent panel
VOCs [7,8,23,25]	Photoactivation, catalytic sensitization, porous analyte access, humidity management, and array-based discrimination when selectivity cannot be achieved with one sensing element	A large response to one VOC may not translate into selectivity in mixtures or under realistic breath and indoor-air humidity	Low-concentration calibration, realistic humidity, interferent mixtures, long-term stability, and multivariate discrimination performance
Humidity [26,37]	Controlled hydroxylation, CNT functional-group density, electrode interfaces, and stabilization of the resistive or impedance baseline	Water adsorption and protonic transport can dominate the signal and mask the intrinsic response to the target gas	Humidity-only controls, dynamic RH cycling, hysteresis, impedance frequency response, and recovery completeness
O_2_ [24]	Photoactivated or dye-sensitized TiO_2_–CNT interfaces with controlled light absorption and efficient interfacial charge transfer	The response may depend simultaneously on oxygen partial pressure, sensitizer stability, wavelength, and illumination intensity	Calibration against oxygen concentration with explicit wavelength, intensity, reversibility, and long-term photostability data

**Table 12:** Representative commercial baselines and their implications for TiO_2_–CNT gas-sensor development.

Technology	Representative Commercial Baseline	Established Strength	Limitation/Benchmark for TiO_2_–CNT
Conventional heated MOS [94]	Figaro TGS2600: 1–30 ppm H_2_ nominal range; 210 mW heater power.	Simple, compact chemiresistive architecture with mature commercial deployment.	Heater power and environmental cross-sensitivity remain relevant; a hybrid should show a measurable system-level gain under comparable concentration and humidity conditions.
MEMS MOS [95,96]	TGS8100: 1–30 ppm H_2_, 15 mW heater. BME688: 320 °C heater target; duty-cycled modes reduce average current but trade power against response time.	Miniaturization, duty cycling, and low average power are already commercially achievable.	Room-temperature sensing alone is not a sufficient advantage; total power, speed, calibration, and robustness should be compared with modern MEMS baselines.
Catalytic bead (pellistor) [97]	SGX VQ548MP: catalytic element at about 400–500 °C; 3.0 V and 34–42 mA; T_90_ < 12 s at 1% methane.	Fast combustible-gas detection in the percent/LEL regime and compatibility with certified safety instrumentation.	Catalyst poisoning and thermal demand are important limitations; a TiO_2_–CNT alternative must also demonstrate durability, poisoning tolerance, response speed, and calibration stability.

**Table 13:** Minimum durability and reliability validation for application-oriented TiO_2_–CNT gas sensors.

Stressor	Failure Mechanism to Resolve	Minimum Validation	Practical Mitigation/ Design Response
Humidity [7,26,37]	Competitive adsorption, hydroxylation, protonic transport, and CNT-junction/ work-function modulation.	Humidity-only controls; analyte tests at several RH values; RH cycling; hysteresis; baseline and recovery tracking.	Humidity conditioning, selective inlet/overlayers, RH reference channel, calibration or algorithmic compensation.
Poisoning/inhibition [97]	Strong or irreversible adsorption on active/catalytic sites; blocked gas access; persistent baseline or sensitivity loss.	Application-relevant sulfur/siloxane challenge where appropriate; purge/regeneration test; pre/post electrical and surface characterization.	Protective or selective filters, regeneration protocol, catalyst/oxide controls, and explicit end-of-life criteria.
Thermal and damp-heat stress [98,101]	Contact drift, morphology or phase evolution, functional- group changes, package/ substrate degradation.	Temperature/RH cycling; qualification-style 85 °C/85% RH where compatible; post-stress response, kinetics, resistance, noise, and structure.	Lower thermal budget, stable contacts, compatible substrate/encapsulation, and application-specific stress limits.
Long-term drift and batch variation [99,100]	Aging, interface reconstruction, process variability, and calibration drift.	Multiple devices from independent batches; statistical dispersion; repeated calibration over weeks/months or justified accelerated aging.	Process controls, reference channels, drift correction, recalibration schedule, and reporting of device-to-device spread.
Mechanical fatigue [16,27]	Cracking, delamination, electrode damage, and permanent change of CNT percolation or TiO_2_–CNT contacts.	Defined bend radius and cycle count; baseline resistance plus gas response before/after fatigue; post-fatigue microscopy or mapping.	Strain-tolerant interlaced networks, neutral-axis placement, robust electrode interfaces, and mechanical design matched to use.

**Table 14:** Methodological gaps in TiO_2_–CNT gas-sensor studies.

Issue	Consequence	Recommended Reporting
Different response definitions [3,5]	Limits cross-study comparison and can obscure the direction and magnitude of the response in mixed oxide/CNT conduction regimes	Explicit response equation, direction of resistance change, baseline convention, response units, and recovery criterion
Humidity not standardized [7,26,37]	Masks the intrinsic analyte response and may create apparent selectivity in oxidized CNT or hydroxylated TiO_2_ surfaces	Relative humidity during baseline and analyte exposure, humidity-only controls, dynamic RH cycling, and humidity- dependent response and recovery
Missing control devices [3,5,47]	Makes it difficult to separate contributions from TiO_2_, CNTs, secondary additives, electrodes, processing conditions, and the true hybrid interface	TiO_2_-only, CNT-only, physical-mixture, additive-only, and true hybrid devices fabricated and tested under comparable conditions
Insufficient mechanistic validation [9,31,35,48]	Weakens causal interpretation of charge transfer, vacancy effects, heterojunction formation, catalytic spillover, or photoactivation	Independent evidence from in situ or operando spectroscopy, XPS, Raman spectroscopy, EPR, impedance, Kelvin-probe measurements, photoconductivity, or noise analysis
Limited stability and reproducibility testing [3,5,27]	Overestimates practical readiness and may conceal drift, poisoning, incomplete recovery, aging, or fabrication variability	Repeated cycles, long-term baseline stability, storage stability, batch-to-batch variability, statistical dispersion, and post-test structural characterization
Incomplete structural reporting [10,11,15,32]	Obscures structure–performance relationships and prevents reproduction of the active TiO_2_–CNT interface	TiO_2_ phase and crystallinity, shell thickness, oxide distribution, CNT type, purification and functionalization routes, dispersion method, film thickness, electrode geometry, and oxide loading
Narrow selectivity tests [5,26,27]	Inflates perceived performance and hides interference from water, oxygen, VOC mixtures, oxidizing gases, or reducing gases	Broad interferent panels under realistic humidity, concentration, temperature, carrier-gas, and flow conditions
Overuse of analogical evidence [3,4,5]	Transfers mechanisms from photocatalysis, photoelectrochemistry, TiO_2_–graphene, or other adjacent systems without demonstrating that they control the response of a TiO_2_–CNT gas sensor	Explicit distinction between direct gas-sensing evidence, supporting interfacial evidence, and contextual mechanistic analogues

Note: The methodological recommendations are the authors’ synthesis of the cited literature. References are placed directly beside each recurring gap so that the evidential basis of each recommendation is explicit.

## Data Availability

No new data were created or analyzed in this study. Data sharing is not applicable to this review article.
